# Formulation of a novel antibacterial topical treatment based on Magnetite-Buforin-II-silver nanobioconjugates

**DOI:** 10.3389/fbioe.2022.1003004

**Published:** 2022-10-28

**Authors:** Laura N. Muñoz, Valentina Jaramillo, Mónica Gantiva-Diaz, Javier Cifuentes, Carolina Muñoz-Camargo, Juan C. Cruz, Andrés Fernando González Barrios

**Affiliations:** ^1^ Grupo de Diseño de Productos y Procesos (GDPP), Departamento de Ingeniería Química y de Alimentos, Universidad de los Andes, Bogotá, Colombia; ^2^ Grupo de investigación en Nanobiomateriales, Ingeniería Celular y Bioimpresión (GINIB), Departamento de Ingeniería Biomédica, Universidad de los Andes, Bogotá, Colombia

**Keywords:** nanobioconjugates, antimicrobial peptide, BUF-II, direct emulsions, stability, biocompatibility, antibacterial activity

## Abstract

Community acquired infections caused by Meticillin-resistant *Staphylococcus aureus* (MRSA) have become a growing concern due to its impact on the world public health. This microorganism is a commonly spreading pathogen associated predominantly with skin infections and connected to other more severe conditions (septic shock, and generalized infection). The lack of highly effective antibiotics and treatments to control skin infections with *S. aureus* has led to the search of novel therapies using alternative agents such as antimicrobial peptides (AMPs). In order to obtain a viable administration route to counteract superficial skin infections (impetigo, abscesses, furuncles, and cellulitis), a topical formulation based on Magnetite-Buforin-II-silver nanobioconjugates as active antibacterial agents was designed by their dispersion in O/W concentrated emulsions. The prepared topical characterization indicated that O/W emulsions were stable in time, the droplets size remained within the appropriate values (∼1 µm) and their rheological properties, such as pseudoplastic and shear-thinning behavior, remained unchanged for up to 3 months. Additionally, hemolysis and platelet aggregation tests were acceptable (i.e., 14.72 ± 2.62% and 8.06 ± 2.90%, respectively) in compliance with the ISO-10993 standard. Furthermore, the treatment reduced significantly (*p <* 0.0001) the growth of both clinical isolated MRSA and wild Type *S. aureus* strains as evidenced by the contact diffusion method. These results are important in the context of proposing new alternatives that allow manage effectively the threat posed by the antibiotic resistant bacterial strains, which jeopardize the lives of thousands of people every year.

## 1 Introduction

Despite bacteria being ubiquitous microorganisms, and just a small percentage of them cause infections, there is a persistent concern due to the increase and prevalence of antibiotic resistance that impact on public health at a global scale (Frieden, 2013; Centers for Disease Control and Prevention, 2020a; Centers for Disease Control and Prevention, 2020b). This impact is related to the fact that there is an increasing level of antibiotic resistant bacteria and in this case, therapeutic options available are quite limited. Specifically, *Staphylococcus aureus* Methicillin Resistant (MRSA resistant to all available penicillin and almost all *β*-lactam antibiotics) is the leading cause of skin infections (Alkiza et al., 2004; Doron and Gorbach, 2008; Ki and Rotstein, 2008; Stevens et al., 2014) in nosocomial infections as well as in community settings (Vingsbo Lundberg and Frimodt-Møller, 2013; World Health Organization, 2020). The severity of the infection in the skin is classified according to the presence of impetigo (minor infection) and eczema, ulcers, as well as lacerations (secondary infection). Skin infections are typically treated in outpatient strategy that includes an oral and topical antibiotics regime. MRSA topical treatments have been based principally in fusidic acid and bacitracin antibiotics (Stevens et al., 2014). However, in the majority of cases these two antibiotics fail to combat MRSA (Bessa et al., 2016). Furthermore, systemic options in severe skin and soft tissues MRSA infections are Vancomycin and Linezolid (Vingsbo Lundberg and Frimodt-Møller, 2013). This highlights the pressing need for new and more effective therapies for the treatment of MRSA skin infections. Moreover, MRSA has been considered a priority in intrahospital environments for the search of more potent antibacterial alternatives (Watkins et al., 2019; Wu et al., 2019; Miller et al., 2020).

A potential alternative for the innovative design of topical treatments are antimicrobial peptides (AMPs), which offer the possibility of eradicating infections with minimal safety issues and the absence of resistance issues in the long-run (Woodburn et al., 2020). Naturally occurring AMPs have been recognized as promising candidates as they are widely distributed in all kingdoms of nature going from bacteria to humans. AMPs are mainly cationic, amphipathic and, among others, exhibit diverse biological activities such as antibacterial (Mishra et al., 2017), antiviral (Agyei et al., 2017), antifungical (Agyei et al., 2017), cytotoxic (Castan˜eda-casimiro et al., 2009), wound healing (Silva et al., 2015) and immunomodulatory (Roberts et al., 2017). An attractive AMP due to its unique properties is Buforin II (BUF-II), which is a frog-derived AMP of 21 amino acids (TRSSRAGLQFPVGRVHRLLRK). BUF-II was first isolated from the frog *bufo gargarizans* stomach Mun˜oz and Groot, 2015), and more recently from the frog *Sphaenorhynchus lacteus* skin (Conlon et al., 2014; Mun˜oz-Camargo et al., 2018). BUF-II has cationic charge (6+), an alpha helix secondary structure, and the ability to penetrate cell membranes without disrupting them. In this regard, BUF-II stands out for its unique an effective antimicrobial mechanism that involves membrane translocation to interact with DNA and ultimately interrupt bacterial replication (Park et al., 2000; Mun˜oz-Camargo et al., 2018). Despite the potential of BUF-II, a major drawback is its low *in vitro* and *in vivo* stability and lifespan, which limits the possibility of considering it for clinical applications (Perez et al., 2019).

These shortcomings have been addressed by a number of strategies including functionalization with polyethylene glycol (PEG) (Perez et al., 2019), entrapment in mesoporous materials (Nordstro¨m et al., 2019), encapsulation within polymeric capsules (Uhrich et al., 1999), liposomes (Ghosh Dastidar and Chakrabarti, 2019), and immobilization on nanomaterials (Bharathala and Sharma, 2019). In this regard, a number of studies have demonstrated that by interfacing AMPs with nanomaterials, such as silica, gold, silver and carbon nanoparticles, their stability and lifespan is considerably increased (Du and Stenzel, 2014; Elahi et al., 2018; Sharma, 2019). This success has been mainly attributed to unique properties such as high surface to volume ratio, size, chemical reactivity, and biological mobility and stability (Murthy, 2007; Nasimi and Haidari, 2013; Bharathala and Sharma, 2019). Recent successful examples include immobilization of the AMP esculentin-1a on gold nanoparticles (Casciaro et al., 2017), the Andersonin-Y1 peptide on silver nanoparticles (Makowski et al., 2019) and the indolicin AMP on carbon nanotubes (Pradhan et al., 2017). Magnetite nanoparticles (MNPs) have also consolidated as one of the most attractive nanoplatforms for immobilization of different biomolecules such as epidermal growth factor (Enriquez-Ochoa et al., 2020), albumin (Maltas et al., 2011), and bacitracin (Byzova et al., 2020). In addition, they exhibit strong magnetic responsiveness that has proven useful to guide their fate and transport, and to facilitate localized thermal energy release and pharmacological targeting (Murthy, 2007). Silver nanoparticles have gained attention because of their excellent antibacterial activity, which made them attractive as additive for numerous products of biomedical interest (Philip, 2010; Kumar et al., 2018). Nonetheless, these nanoparticles have been reported to be highly cytotoxic alone and therefore biosynthesis schemes (Kumar et al., 2018) or co-assembly with other nanostructured systems (Ram´ırez- Acosta et al., 2020) have emerged as strategies to potentiate their use in medical treatments without major biocompatibility issues.

Considering the limited number of topical treatments options commercially available to counteract skin MRSA infections, the aim of this work was to synthesize and characterize novel topical treatments based on magnetite nanoparticles, PEG8, Buforin II, and silver (MNP-PEG8-BUF-II-Ag) nanobioconjugates, which were designed as a strategy to not only extend BUF-II’s stability, activity, and lifespan but to synergistically incorporate the antibacterial potency of silver (Cuellar et al., 2018). Furthermore, the MNP-PEG8-BUF-II-Ag nanobioconjugates were dispersed into O/W emulsions to form the topical treatment, which was characterized in terms of stability, rheological response, and particle size. Here, we determined the antibacterial capacity of the nanobioconjugates and topical treatments against MRSA as well as their biocompatibility *in vitro*. Finally, we conducted a scaling-up experiment to assure reproducibility of the topical production at the bench scale.

## 2 Materials and methods

### 2.1 Materials

Iron (III) Chloride Hexahydrate (97%), Tetramethylammonium Hydroxide (TMAH) (25%), (3-aminopropyl) Triethoxysilane (APTES) (98%), N-hydroxy Succinimide (NHS) (98%), N-[3- dimethylammino)-propyl]-N′-ethyl Carbodiimide Hydrochloride (EDC) (98%), Glutaraldehyde (25%), de (NHS) Mineral Oil (99.0%), Tween 20 (Polysorbate 20) (GC grade), Tween 80 (Polysorbate 80) (GC grade), Span 80 (Sorbitan Monooleate) (GC grade), Thrombin, Triton X-100 (Laboratory grade), Sodium Alginate (Pharmaceutical grade) and MTT Formazan were purchased from Sigma-Aldrich (Milwaukee, WI). Dimethyl sulfoxide (DMSO) (99.0%) was obtained from Sigma-Aldrich (Allentown, PA) and Silver Nitrite (AgNO_3_) (99%), NH2-PEG8-Propionic Acid (98%), Carbopol (poly (acrylic-acid)) (*>*99.0%) and Cytotoxicity Detection Kit (LDH) were purchased from Sigma-Aldrich (St. Louis, MO). Iron (II) Chloride Tetrahydrate (98%), dimethylformamide (DMF) (99.8%), Sodium Chloride (NaCl) (99.9%), Sodium Hydroxide (NaOH) (98%) and Triethanolamine (*>*99.0%) were obtained from PanReac AppliChem (Spain). Dulbecco’s Modified Eagle’s Medium (DMEM), Fetal Bovine Serum (FBS) and Trypsin-EDTA (1X in PBS) were purchased from Biowest (Nuaille’, FR). Penicillin/Streptomycin (P/S) was obtained from Lonza. Mueller Hinton Agar and Lysogeny Broth (LB) for microbiological test were obtained from Merck-Millipore (Darmstadt, GER) and Merck-Millipore (Milwaukee, WI), respectively. LIVE/DEAD™ BacLight™ Bacterial Viability Kit L13152 were purchased from Thermo Fisher Scientific (Eugene, OR). Buforin II (BUF-II) was synthesized by GL Biochem Shanghai (Shanghai, China) and the Peptide Synthesis Facility at Pompeu Fabra University (Barcelona, Spain). Bacteria strains were clinical MRSA isolates (92250621: arm skin secretion, 93040389: knee skin secretion and 93190573: hand skin secretion) collected by Fundacio´n Cardioinfantil and wild type *S. aureus* (ATCC 23235) and *E. coli* (ATCC 25922) were purchased from ATCC (St. Cloud, MN, United States). Cytotoxicity assays were conducted with HaCaT (ATCC® CRL-2404) cells.

### 2.2 Synthesis and characterization of MNP-PEG8-BUF-II-Ag nanobioconjugates

A mixture of 1 g of FeCl_2_·4H_2_O and 2.71 g of FeCl_3_·6H_2_O was dissolved separately in 5 ml of distilled water to obtain 1 and 2 M solutions, respectively. Chloride solutions were then mixed and heated up until a homogeneous solution was obtained. When the solution reached 80°C, 2 ml of 2% (v/v) solution of Tetramethylammonium Hydroxide (TMAH) was added. At the same time, 1.6 g of NaOH were dissolved in 5 ml of type I water (Ultrapure water, resistivity 18 MΩ -cm, conductivity 0.056 μS/cm) (8 M solution), and this solution was also heated to 80°C. The hot NaOH solution was finally dropped at 200 μl/min into the chloride mixture under vigorous stirring at 1500 RPM. The reaction was carried out for 1 h at 80°C under continuous stirring at 1500 RPM. The obtained MNPs were washed at least 3 times with distilled water with the aid of a strong permanent magnet, and sonicated after each wash (2800 ultrasonic cleaner, Branson, MO, United States) for 5 min at 40 kHz frequency (Perez et al., 2019).

For surface functionalization, 50 ml of magnetite nanoparticles solution (2 mg/ml) was sonicated vigorously until complete homogenization. In total, 2 ml of 2% (v/v) solution of TMAH, 50 µl of 99% glacial acetic acid and 100 µl of 10% (v/v) APTES were added to resuspend the MNPs. The sample was then kept for 1 h at 60°C and 250 RPM to carry out the chemical reaction. Finally, the sample was washed several times with 1.5% (w/v) NaCl saline solution and type I water to remove excess reagents. The separation process in between washes was aided by a strong neodymium permanent magnet.

To modify the surface of the nanoparticles and confer flexibility to the immobilized molecules, a PEG-8 polymer was conjugated to the silanized nanoparticles obtained previously. Briefly, 50 ml of silanized nanoparticles (2 mg/ml) were mixed with 1 ml of 2% (v/v) glutaraldehyde solution and left to react for 30 min. Next, the polymer NH_2_-PEG8-Propionic acid solution was added and stirred overnight, followed by several washes with type I water. A mixture of EDC/NHS, pre-dissolved in DMF, and 500 µl of BUF-II solution at 1 mg/ml in sterile NaPB were added to 100 mg of PEG8-coated magnetite nanoparticles suspended in 30 ml of distilled water by sonication. After 5 min of sonication and then, 24 h of reaction, the obtained MNP-PEG8-BUF-II nanobioconjugates were thoroughly washed with type I water with the aid of a strong magnet to remove reagents excess.

To boost the antimicrobial effect of the MNP-PEG8-BUF-II nanobioconjugates, see Supplementary Figure S1, a silver core shell was synthesized on the surface of the nanobioconjugates using Silver Nitrite (AgNO_3_) as precursor. Additionally, honey was used as the reducing and stabilizing agent in the synthesis of silver shells. In this process, the glucose in honey serves as the main component for reduction of silver ions (Ag^+^) and subsequently, stabilizes the reduced silver (Ag^0^) to form the Ag shell. Moreover, additional minor components of honey such as proteins and peptides, improve the stabilization of the silver shells acting as capping agents. Briefly, 100 ml of 1 mg/ml nanobioconjugates solution in type I water was sonicated followed by addition of AgNO_3_ to reach a final concentration of 10^−3^ M. The resulting solution was then sonicated in an ultrasonic bath (2800 ultrasonic cleaner, Branson, MO, United States) for 5 min and then magnetically stirred at 600 RPM. 75 ml of honey solution were then incorporated into the nanoparticles solution followed by magnetic stirring for 5 more minutes. The pH (pH 3) was then rapidly elevated to 8 with NaOH (5 M) under agitation at 600 RPM for 1 h at room temperature. The obtained MNPsPEG8-BUF-II-Ag nanobioconjugates were precipitated with the aid of a strong magnet and thoroughly washed with type I water. Then, the nanobioconjugates were centrifuged at 4000 RPM for 5 min to remove the reagents excess and, finally, resuspended in type I water (2 mg/ml), sonicated to avoid aggregation, and stored at 4°C until further use.

### 2.3 Oil in water (O/W) emulsions preparation

O/W emulsions were prepared with two different concentrations of dispersed phase (60% wt. and 70% wt.) and varying the hydrophilic-lipophilic balance (HLB) value (11.79 and 14). Table 1 shows the components and corresponding amounts of a typical O/W formulation (Wibowo and Ng, 2001). For benchmarking purposes, we included a commercially available antimicrobial topical treatment, which contains fusidic acid as the active antimicrobial component. Triethanolamine and Tween 80 were added to distilled water, whereas Span 80 was dissolved in Mineral Oil. Each phase was then filtered with cellulose (0.22 µm pore size, GE Healthcare, UK) and PTFE (0.45 µm pore size, FOXX Life Sciences, US) filters, respectively. The solid thickener, Carbopol, was placed over a glass petri dish and then exposed to germicidal UV light for 15 min.

**TABLE 1:** List of reagents and composition of the bare O/W emulsion at two different concentrations of the dispersed phase and two different HLB values: 60 % wt. and 70 % wt. and HLB of 11.79 and 14.

Composition of O/W emulsions (%)				
	60% wt		70% wt	
Reagents	HLB 11.79	HLB 14	HLB 11.79	HLB 14
Water	36.34	35.54	26.34	25.54
Triethanolamine	0.43	0.43	0.43	0.43
Tween 80	2.8	3.6	2.8	3.6
Carbopol	0.43	0.43	0.43	0.43
Mineral Oil	58.8	59.6	68.8	69.6
Span 80	1.2	0.4	1.2	0.4

An aqueous suspension of MNP-PEG8-BUF-II-Ag nanobioconjugates (1 mg/ml) was formed by vigorous vortex agitation followed by 5 min of agitation in an incubator shaker. The aqueous phase of the emulsion was stirred at 250 RPM while adding the nanobioconjugate suspension dropwise. Carbopol was then added slowly to the aqueous phase under vigorous agitation. Next, the oil phase was incorporated into the aqueous phase with the aid of a peristaltic pump G1000 (Fisherbrand™, Madrid, ES) operating at 20% of its maximum velocity. The mixture was maintained under vigorous stirring at 800 RPM with an overhead stirrer Hei-TORQUE Precision 400 (Heidolph, Schwaback, GE) equipped with a 60° pitch-blade impeller until the oil phase addition was completed. To obtain a stable emulsion, the mixture was then agitated at 1200 RPM for 10 more minutes at room temperature. The obtained emulsions were bottled in glass flasks and stored at room temperature until further use.

### 2.4 Emulsion stability measurements

O/W emulsions were visually inspected for the first 3 days after preparation every 24 h to evaluate for the presence of phase separation, destabilization phenomena or any other change in appearance. After this period, emulsions were stored for 90 days in a stability chamber (RGX—250E, THOMSON) at 40°C and 75% humidity. Additional to visual inspection, we monitored the Turbiscan Stability Index (TSI) daily with cumulative scans in a Turbiscan instrument (FORMULACTION SAS, L’Union, FR), which provides information regarding possible destabilization phenomena of a given sample (SAS, F., 2014). Furthermore, the same instrument allowed us to obtain information on the transmittance and backscattering percentage as additional indicators of instability phenomena. For the measurements, samples were transferred to a glass vial at room temperature while avoiding bubble formation at the top of the container. The collected scans were plotted together for comparison.

### 2.5 Particle size analysis of droplets in emulsions

A Mastersizer 3000 instrument (Malvern Instruments Ltd., Worcestershire, UK) was used to determine average particle size and particle size distribution of droplets in the emulsions (Malvern, 2017). This was accomplished by averaging 5 measurements per sample. In direct emulsions, the selected dispersant was water with a refractive index of 1.33 while the material was Mineral Oil. Obscuration lower and upper limits were fixed at 10%–20% for O/W emulsions.

### 2.6 Rheological characterization of emulsions

Rheology characterization of the emulsions was conducted with a DHR1 rheometer (Discovery Hybrid Rheometer, Ta Instruments, DE, US). Flow and oscillatory sweep experiments were performed to determine the rheological behavior of the emulsions and to assess changes in their storage (G′) and loss (G″) moduli. All experiments were performed with a parallel plate geometry (20 mm) at 20°C. Flow sweep was performed between 0.1 and 200 s^−1^ (Lauterbach and Mu¨ller-Goymann, 2014). Additional information about thixotropy was obtained by a second flow sweep beTween 200 and 0.1 s^−1^. The stress vs. shear-rate data was fit to the power law model (Eq. 1) (Wibowo and Ng, 2001).

$$\tau =m{\overset{\cdot }{\gamma }}^{n}$$ (1)

*τ* is the stress, *γ*˙ is the shear rate, *m* is the consistency index, and *n* is the flow index.

The oscillatory test in frequency was performed at 1% strain for frequencies between 0.01 and 1 Hz.

### 2.7 Scaling-up process of the emulsion at bench scale

The production of the emulsion was further carried out at the bench scale to evaluate whether the developed process can be scaled-up to produce 3,000 g of the direct emulsion. Accordingly, it was necessary to consider some scaling-up parameters based on the initial assembly to assure the same synthesis operation conditions (Supplementary Table S1). The followed geometrical ratios for the design were obtained with the aid of Supplementary Equation S1. The impellers were designed according to the required dimensions in Autodesk® Inventor® and then 3D printed in a Ultimaker 2+ 3D printer (Utrecht, Netherlands) (Supplementary Figure S2).

For the mixing and homogenization process, the tip velocity was kept constant during the scaling-up, and Eq. 2 was followed to calculate it:

$$V_{t}=\pi DN$$ (2)

*V*_*t*_ is the tip velocity, *D* is the impeller diameter and *N* is the fixed velocity in RPM.

### 2.8 Antibacterial assays

The bacteria strains employed in this assay were methicillin resistant strains of *Staphylococcus aureus* (MRSA) 92250621, 93040389 and 93190573, corresponding to clinical isolates from skin secretion samples collected from the arm, knee, and hand. For reference, we also evaluated *S. aureus* (ATCC 23235) and *E. coli* (ATCC 25922). To semi-quantitatively assess the antibiotic resistance of the MRSA strains, a disk susceptibility test (i.e., antibiogram) was performed using antibacterial susceptibility discs of methicillin (5 µg), ampicillin (10 µg), gentamicin (10 µg) and vancomycin (30 µg). For this purpose, MRSA and *S.aureus* strains were grown to 1 × 10^7^ CFU. Subsequently, a mass culture was performed in Mueller Hinton agar plates and the antibacterial susceptibility discs were placed in punch holes made over the agar. The plates were incubated at 37°C for 24 h. The antibiogram for each strain was performed in triplicate, see Supplementary Figure S3. The inhibition halo was measured aided by the image processing software ImageJ® and the results were interpreted as sensitive, intermediate, or resistant according to the CLSI-NCCLS reference standards.

The antibacterial activity of the topical treatments was assayed by direct contact following a modification of the disk susceptibility test described by the standard M01-A11 (Clinical and Laboratory Standards Institute, 2012). Briefly, bacterial cells from MRSA clinical isolates strains, *S. aureus* (ATCC 23235) and *E. coli* (ATCC 25922) were cultured and aliquoted to reach 1 × 10^6^ CFU. 500 µL of each treatment, namely O/W emulsion with MNPs-PEG8-BUF-II-Ag (400 μg/ml), nanobioconjugates, O/W emulsion without nanobioconjugates, and commercial fusidic acid topical treatment (20 mg/ml) were dispensed, under sterile conditions, in 2 ml microcentrifuge tubes in triplicate. Subsequently, 500 µl of the 1 × 10^6^ CFU bacteria suspension were exposed to the treatments (previously transferred into microcentrifuge tubes) for 2 h at 37°C. Bacteria grown (51 × 10^6^ CFU) in Na_2_HPO_4_ buffer were employed as positive control. Serial dilutions (1:10) of the grown bacteria, after the exposure to the treatments in Na_2_HPO_4_ buffer were prepared starting from 100 µL of the supernatant for each treatment. Then, 100 µl of the third dilution were plated in Mueller Hinton agar plates (diameter of 90 mm) and incubated for 18 h at 37°C. The colony forming units (CFU) were counted for each treatment and the bacterial growth percentage was then calculated by considering the used dilution factor. Bacteria exposed to Na_2_HPO_4_ buffer was employed as positive control (i.e., bacterial growth of 100%). An ANOVA one way followed by a t-student were performed as statistical analysis of the data. The results were considered significantly different when *p <* 0.05.

### 2.9 Wound infection assay *ex vivo*

Porcine skin was obtained from a local butcher shop and the fatty tissue was carefully removed by mechanical means. Squared samples (1 cm side) of the tissue were cut and a 0.5 cm length superficial wound was made aided by a scalpel. The samples were placed on Petri dishes and washed twice with sterile PBS (1X). Subsequently, 2 µl of a 1 × 10^8^ CFU *S. aureus* (ATCC 23235) solution was inoculated on the wound along with 2 µl of LB media. The *ex vivo* infected skin was incubated for 72 h at 37°C with 10 ml of PBS (1X) and replaced daily to maintain hydration. After incubation, the infected wound was exposed to 50 µl of the treatment (O/W emulsion with 400 μg/ml of MNPs-PEG8-BUF-II-Ag nanobioconjugate) and PBS (1X) dispensed with the aid of an insulin syringe and carefully spread on the wound with a spatula. The infection was inspected in time by macroscopic visualization. The effect of the treatments on the infection progress was monitored through confocal microscopy. For the observation under confocal microscopy, the LIVE/DEAD™ BacLight™ Bacterial Viability Kit (L13152) was employed to differentiate live and dead bacterial cells from the porcine tissue.

### 2.10 Biocompatibility assays

#### 2.10.1 Hemolysis assay

Hemocompatibility was assessed by following the ISO 10993-4 standard (Standard, I., 2002; ISO, B. S. E. N., 2006; U.S. Department of Health and Human Services Food and Drug Administration, 2016) for biological evaluation and the ISO 10993–12 (ISO, B. S. E. N., 2006) standard for sample preparation and reference materials. A sample of 100 µl of the treatments (O/W emulsion with MNPs-PEG8-BUF-II-Ag nanobioconjugates), O/W emulsion without the nanobioconjugates, and the commercial fusidic acid topical control were deposited into a 96-well microtiter plate and centrifuged at 1800 RPM for 5 min to assure a flat and homogeneous surface for each treatment (six replicates).

The blood sample was collected from a healthy volunteer in vacutainer blood tubes supplemented with EDTA and then the erythrocytes were obtained following the protocol by Cuellar et al. (2018). The samples were obtained with the approval of the Ethical Committee at the Universidad de los Andes (minute number 928-2018). A 1:10 dilution of erythrocytes (2 × 10^7^) in PBS (1X) was prepared, and then 100 µl were pipetted into a 96-well microplate containing the emulsion samples followed by incubation at 37°C for 1 h. Then, 50 µl of the supernatant were transferred to a brand new 96-well microtiter plate and read at 450 nm in a microplate reader (Multiskan™ FC Microplate Photometer, Thermo Scientific, FIN). The positive control was an erythrocyte suspension incubated with Triton X-100 (100% hemolysis), while the negative control was an erythrocyte suspension incubated with PBS 1X (0% hemolysis).

#### 2.10.2 Platelet activation assay

The effect of emulsions on platelet activation was evaluated using platelet-rich plasma (PRPs) obtained from blood of a healthy donor, which was previously collected in vacutainer blood tubes supplemented with sodium citrate following the protocol by Perez et al. (2019). To obtain a calibration curve two-fold serially diluted concentrations of PRPs were evaluated starting at 100% (v/v) down to 0.050% (v/v). Briefly, 100 µl of each treatment, emulsions prepared in the presence and absence of MNPs-PEG8-BUF-II-Ag nanobioconjugates, and the commercial topical control (fusidic acid) were deposited in a 96-well microtiter plate and then exposed to 50 µL of PRP and incubated for 30 minuntes at 37°C and 5% CO_2_. The unaggregated platelets supernatant was removed, and each treatment was rinsed with PBS (1X) and then exposed for 15 min to 100 µL of Triton X-100. Finally, 50 µL of the supernatant were analyzed *via* a LDH assay and read at 493 nm in a microplate reader (Multiskan™ FC Microplate Photometer, Thermo Scientific, FI) to estimate platelet activation. The platelet activation percentage was obtained by comparing the obtained values for the treatments against a calibration curve. An ANOVA one way followed by a t-student test were performed as statistical analysis of the data of both hemocompatibility tests. The results were considered significantly different when *p <* 0.05.

#### 2.10.3 Cytotoxicity assay

MTT Cytotoxicity assay was performed on extracts of the samples method in compliance with the International Standard ISO 10993-5 (International Organization For Standardization, 2009). To prepare the emulsions extracts, a 3% (v/v) solution of each emulsion (O/W emulsion in the presence and absence of MNPs-PEG8-BUF-II-Ag nanobioconjugates, and the commercial fusidic acid topical control) was prepared using DMEM culture media without SBF and then incubated for 24 h at 37°C. Briefly, each of the obtained extracts was subsequently filtered through cellulose filters (0.22 µm pore size, GE Healthcare, UK). Human keratinocytes (HaCat ATCC® CRL-2404) cells were seeded at a density of 2 × 10^4^ per well in a flat-bottom 96-well microtiter plate and incubated for 24 h at 37°C and 5% CO_2_. The cells were then exposed to 100 µl of each treatment serial dilutions from 3% (v/v) to 0.188% (v/v), in triplicate, and incubated at 37°C and 5% CO_2_ for 24 h. Then, 10 µl of MTT (5 mg/ml) reagent were added to each well and incubated for 2 h at 37°C and 5% CO_2_. Finally, the culture medium was removed, and 100 µl of DMSO was added to dissolve the formed formazan crystals. Absorbance was read at 595 nm in a Multiskan FC microplate reader (Thermo Fisher Scientific, Waltham, MA, United States).

## 3 Results and discussion

### 3.1 Synthesis and characterization of MNP-PEG8-BUF-II-Ag nanobioconjugates

**FIGURE 1:**
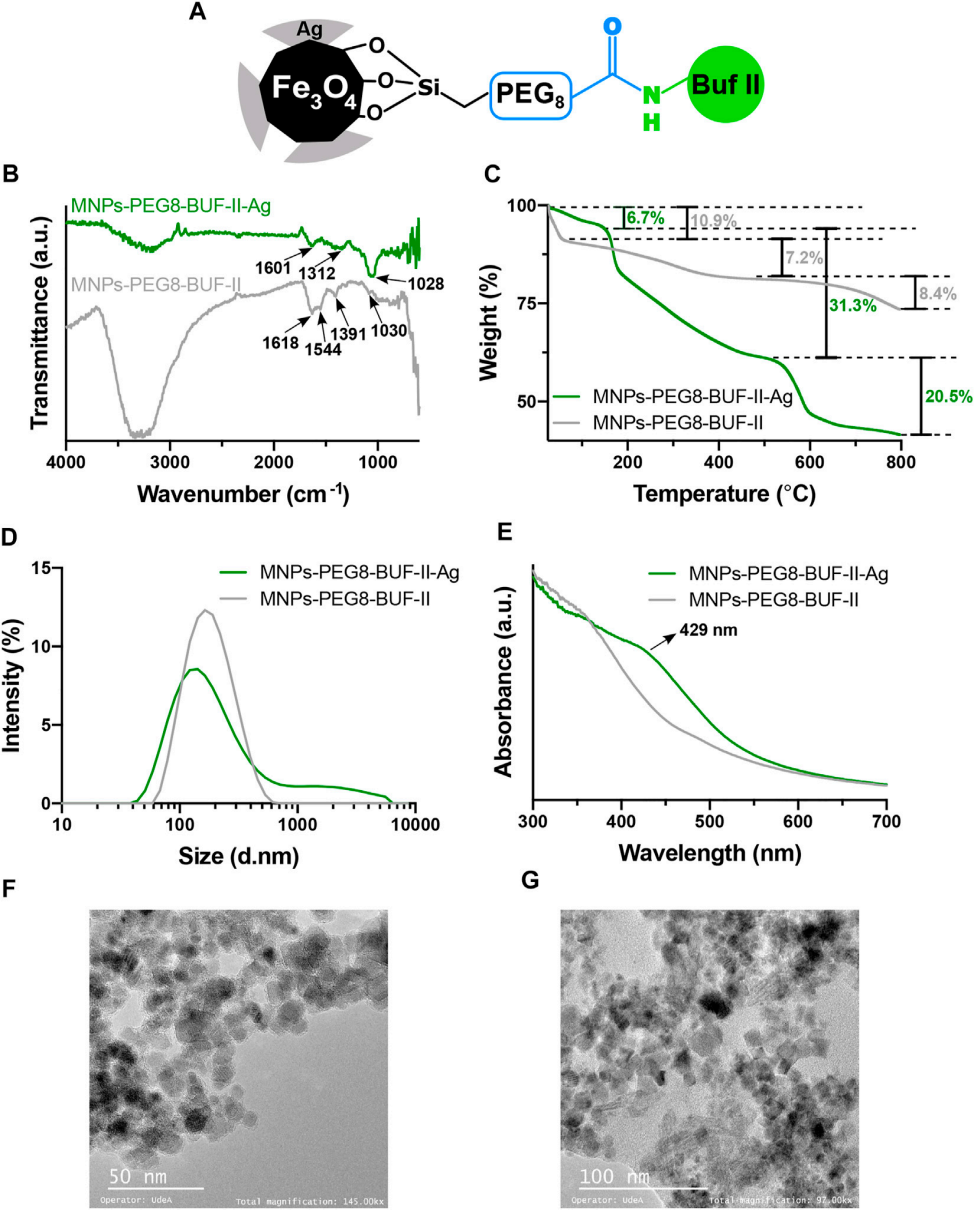
Characterization of MNPs-PEG8-BUF-II-Ag nanobioconjugates. **(A)** Schematic of the nanobioconjugate after the synthesis. **(B)** and **(C)** correspond to the FTIR and TGA analysis, respectively. **(D)** Mean hydrodynamic diameter of the nanobioconjugates *via* DLS. **(E)** UV-VIS analysis of the nanobioconjugate before and after the Ag core-shell growth. The morphology and size of the MNPs- PEG8-BUF-II-Ag nanobioconjugates were analyzed through TEM at two different magnifications: **(F)** 145 kX and **(G)** 97 kX.

Figure 1A shows a schematic representation of the synthetized MNPs-PEG8-BUF-II-Ag nanobioconjugates. In addition, Figures 1B,C show the FTIR and TGA analyses for the MNPs-PEG8-BUF-II and the MNPs-PEG8-BUF-II-Ag nanobioconjugates. The FTIR spectrum of MNPs-PEG8-BUF-II shows peaks at 1,618 and 1,544 cm^−1^, which correspond to the amide I and amide II bands, confirming the correct immobilization of BUF II. Moreover, peaks at 1,391 and 1,030 cm^−1^ can be attributed to C-H and Si-O stretching vibrations associated with the silanization process (Perez et al., 2019). In contrast, the FTIR spectrum of MNPs-PEG8-BUF-II-Ag nanobioconjugates presents three observable peaks at 1,601, 1,312 and 1,028 cm^−1^, which can be mainly attributed to capping and stabilization processes from the synthesis of Ag NPs shell with honey. The peak at 1,601 cm^−1^ can be attributed to the amide I band associated with the capping proteins, while the peak at 1,312 cm^−1^ is attributed to the C-O stretching mode and the very strong peak at 1,028 cm^−1^ is associated to the C–O–C symmetric stretching and C–O–H bending vibrations of proteins present in the honey (Philip, 2010). The obtained thermogram for the MNPs-PEG8-BUF-II agree well with those obtained by us previously where three main weight losses can be identified (Perez et al., 2019). The first one (10.9%, 120°C) is attributed to a dehydration of the samples, while the second one (7.2%, 120–400°C) corresponds to the desorption excess reagents and impurities from the immobilization and functionalization steps. The final loss (8.4%, 400–800°C) is associated with the breakdown of silane covalent bonds and therefore, this weight loss mainly corresponds to the detachment from the surface and degradation of immobilized molecules (APTES, PEG8 and BUF II). The thermogram of the MNPs-PEG8-BUF-II-Ag nanobioconjugates also shows three weight losses. The first one (6.75%, 155°C) can be also associated with humidity loss. However, in this case, the second one is mainly attributed to the desorption of monosaccharides contained in the honey used during the silver deposition (Kumar et al., 2018) and to non-reactive impurities from the reagents employed for the synthesis processes. Finally, the third weight loss is associated to the immobilized molecules and the capping and stabilizing proteins of the honey (Kumar et al., 2018). The final 41.5% remaining weight corresponds to bare MNPs and the Ag NPs shell.

The size and morphology of the nanobioconjugates was studied *via* DLS and TEM. Figure 1D shows the hydrodynamic diameter of the nanobioconjugates. The MNPs-PEG8-BUF-II nanoobioconjugate exhibited a hydrodynamic diameter of 197.98 ± 33.2 nm (PDI = 0.213). This size is significantly lower than that obtained in our previous work (Perez et al., 2019), which can be explained by an increase in colloidal stability after PEG8 conjugation compared with the polyether amine (PEA) used previously as a functional spacer. However, after depositing the Ag shell, the hydrodynamic diameter increased to 443 ± 4.5 nm (PDI = 0.425), which is consistent with our previous findings (Ram´ırez-Acosta et al., 2020). The correct synthesis of the Ag shell was also confirmed *via* UV-VIS. In this regard, Figure 1E shows that the UV-VIS spectrum for the MNPs-PEG8-BUF-II-Ag nanobioconjugates exhibited an increase in absorbance at 429 nm, which has been typically observed for nanometric Ag (Kumar et al., 2018). Figures 1F,G shows TEM images of the MNPs-PEG8-BUF-II and MNPs-PEG8-BUF-II-Ag nanobioconjugates, respectively. Consistent with our previous work, the MNPs-PEG8-BUF-II nanobioconjugates exhibited rounded nanostructures with an average diameters of 9.1 ± 1.6 nm (Perez et al., 2019). The image shows agglomerates of individual nanoparticles with a relatively uniform size and shape. In contrast, MNPs-PEG8-BUF-II-Ag presents an important increase in size, resulting in an average diameter of 15.6 ± 2.2 nm for individual nanoparticles with shapes that lost the sphericity observed initially for the bare MNPs. This can be attributed to the Ag shell growth, which proceeds somewhat uncontrolled on the surface, leading to larger particles with diverse morphologies.

### 3.2 Direct (O/W) emulsions stability

**FIGURE 2:**
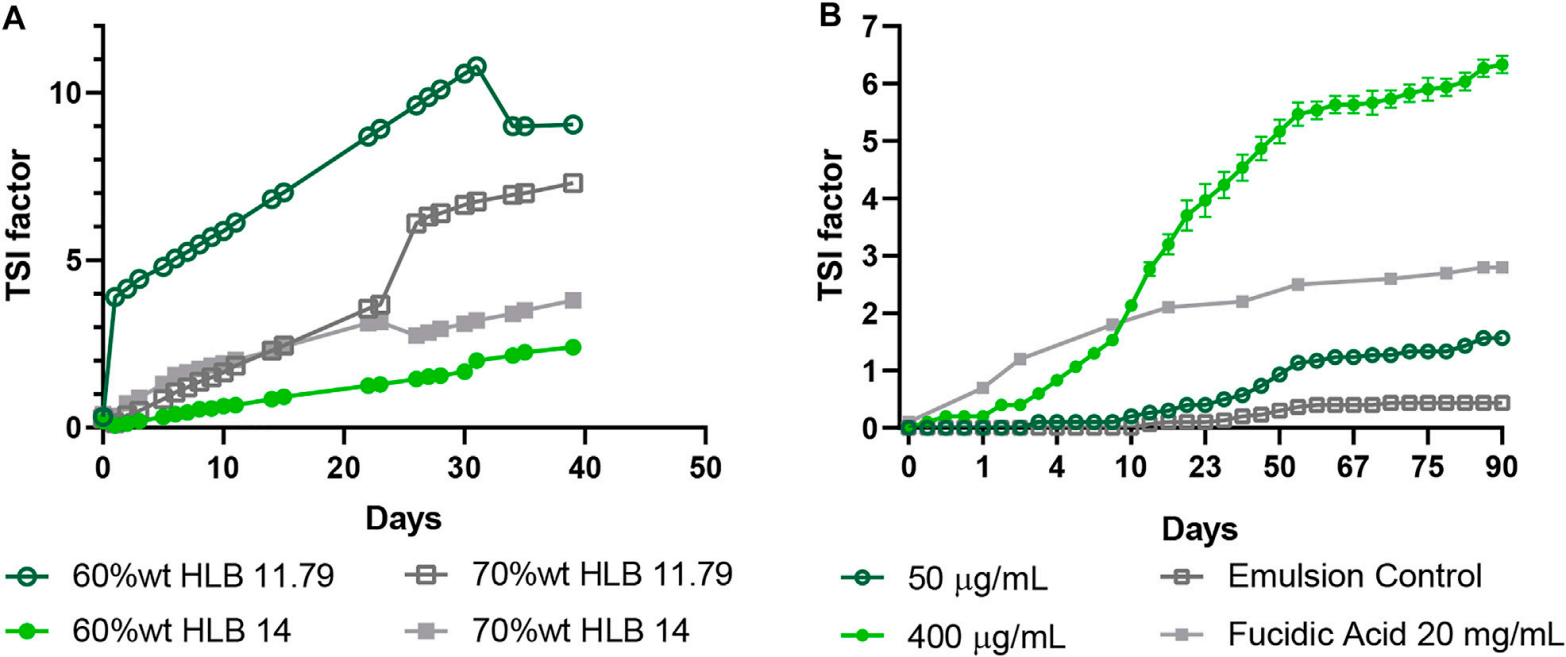
Time evolution of the stability index for bare O/W emulsions as measured in the Turbiscan instrument. **(A)** Emulsions at 60% wt., 70% wt. and, HLB of 11.79 and 14 analyzed for 39 days. **(B)** Emulsions were prepared with the MNPs-PEG8-BUF-II-Ag nanobioconjugates (50 μg/ml and 400 μg/ml) at 60% wt. and HLB 14. The emulsions were analyzed for 90 days after preparation.

O/W emulsions prepared in the absence of the MNPs-PEG8-BUF-II-Ag nanobioconjugates were analyzed in the Turbiscan instruments to determine stability changes. Four O/W emulsions at two dispersed phase concentrations (60% wt. and 70% wt.) and two HLB values (11.79 and 14) were prepared (see Table 1), owing to the high stability reported for concentrated emulsions (Pradilla et al., 2015; Calvo et al., 2020). The emulsions were monitored daily (Figure 2A) to identify possible physical instability phenomena such as flocculation or coalescence. After 39 days of analysis, no visible changes were observed in the samples, and the TSI value remained below 4 for the 14 HLB emulsions. On the contrary, the emulsions with a lower HLB value were considered more unstable due to their TSI values above 5. According to Wibowo and Ng (2001) and Li et al. (2018), it is expected that the more concentrated the emulsions, the higher the stability. This has been attributed to much more small homogeneous drops, which in turn, leads to a more viscous fluid of enhanced stability as reported for colloidal systems containing low concentrations of polymeric thickeners. Nonetheless, this was not the case of the emulsion at 70% wt. and HLB 11.79 and, instead, the emulsion at 60% wt. and HLB of 14 exhibited the lower TSI variation. For this reason, it was selected as the most stable formulation for further analyses.

Maintaining constant the dispersed phase concentration and varying the HLB value allowed us to identify formulations with the required stability, which was related to the fact that non-ionic surfactants with HLB values above 6 favor the formation of O/W emulsions most likely due to a larger exposed hydrophilic surface (Salager, 2002; Rosen and Kunjappu, 2012). In this regard, hydrophilic heads interact easily with polar substances, which favors water solubilization processes (Rosen and Kunjappu, 2012). This was achieved by incorporating Tween 80 (HLB 15) at a higher ratio in the surfactant mixture (7:3), thereby increasing the hydrophilic affinity of the emulsion.

After selecting the most appropriate formulation (60% (w/w) and HLB 14), the emulsion was mixed with varying amounts of dispersed MNPs-PEG8-BUF-II-Ag nanobioconjugates (400, 330, 200, 100, and 50 μg/ml). As observed in Figure 2B, the emulsion with the lowest concentration of MNPs-PEG8-BUF-II-Ag nanobioconjugates (50 μg/ml) showed a TSI variation similar to that of the bare emulsion, albeit at a TSI value of 1.57 ± 0.06 after 90 days. Additionally, the emulsion with the highest amount of MNPs-PEG8-BUF-II-Ag nanobioconjugates exhibited a higher increase of the TSI value (6.33 ± 0.15); however, visual inspection revealed no instability phenomena. Despite the measured TSI values, exposure of the emulsions to accelerated aging conditions (i.e., 40°C and 75% humidity for 90 days) led to no observable instability phenomena. Moreover, the emulsions in presence of the MNPs-PEG8-BUF-II-Ag nanobioconjugates (50 μg/ml) and the bare emulsion exhibited higher stability than that reported for the fusidic acid-based product (TSI value of 3 approximately). Additionally, after 90 days of the preparation, all the emulsions showed a pH close to 7.

These results suggest that the presence of the MNPs-PEG8-BUF-II-Ag nanobioconjugates at concentrations up to 400 μg/ml showed no substantial impact on the stability of the emulsion. This indicates that, despite remaining in the continuous phase of the O/W emulsions, the MNPs-PEG8-BUF-II-Ag nanobioconjugates appear to have little interference with the droplet formation process, which may be explained by their nanoscale size (i.e., diameters of 7.84 nm ± 1.61 nm). This size is about three orders of magnitude lower than that of droplets (mean hydrodynamic diameter of 1.24 ± 0.001 µm). In addition, low concentrations (50 μg/ml) of MNPs-PEG8-BUF-II-Ag nanobioconjugates impede the coalescence of oil droplets; however, as their concentrations increases, instability also increased, favoring coalescence within a few minutes after the homogenization step (data not shown).

### 3.3 O/W emulsions drop size analysis

**FIGURE 3:**
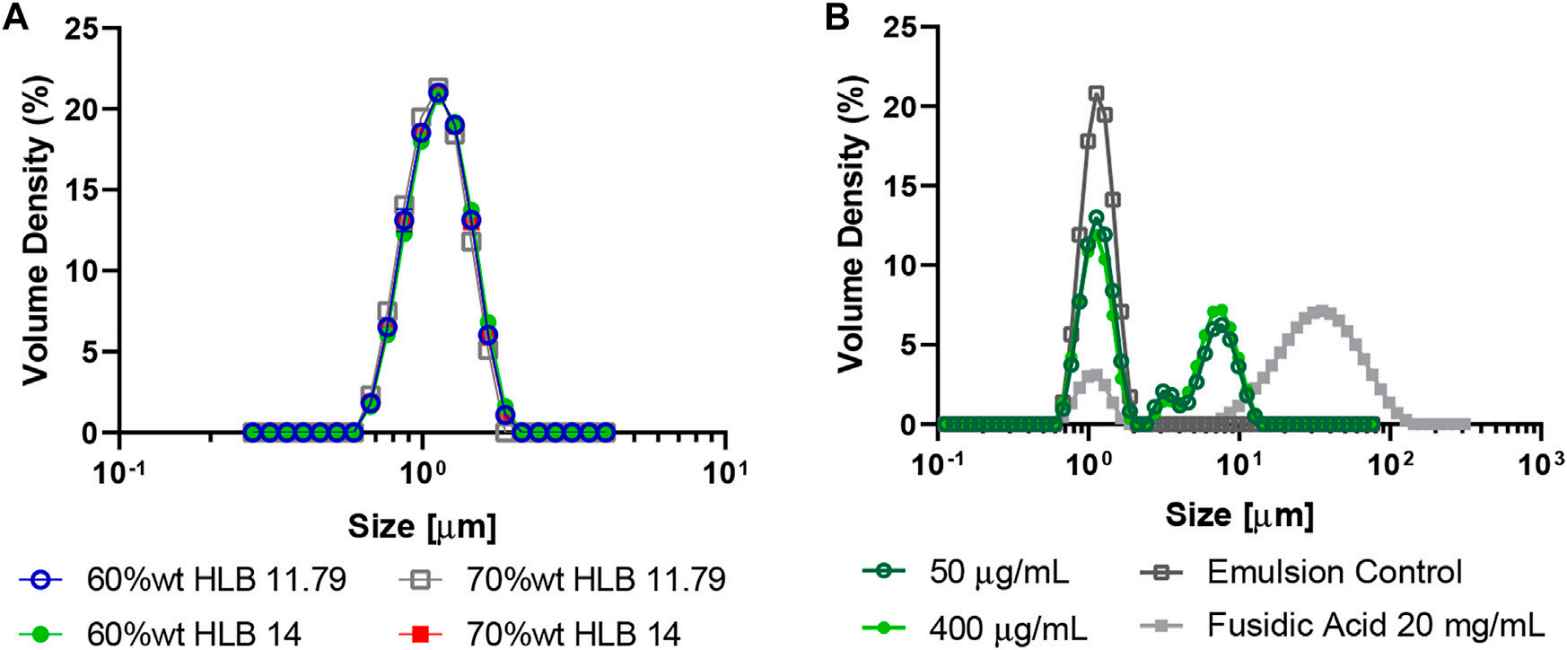
Mean hydrodynamic diameter of O/W emulsions as measured in a Mastersizer 3,000 instrument. **(A)** Emulsions at 60% wt., 70% wt., HLB of 11.79 and 14. **(B)** Emulsions were prepared with the MNPs- PEG8-BUF-II-Ag nanobioconjugates (50 μg/ml and 400 μg/ml) at 60% wt. and HLB 14.

Droplets mean hydrodynamic diameter D[4,3] was measured in a Mastersizer 3,000 instrument (Rawle et al., 2003). Droplets’ size for bare O/W emulsions prepared at different concentrations and HLB values showed a normal distribution (Figure 3A). Here, we obtained a mean hydrodynamic diameter D[4,3] of approximately 1 μm, which is an appropriate droplet size to avoid precipitation or any other instability phenomena (Yang et al., 2019). These results were also observed for all the emulsions even after 95 days.

The favorable results in droplets’ size and their positive impact on the stability of the emulsions may be attributed to the stabilizing role of polymeric matrix formed by the thickener, Carbopol®, in the continuous phase, which impedes coalescence of oil droplets. Carbopol® is one of the most known synthetic thickeners and it is often used in emulsified systems thanks to the imparted sensorial properties, but also by the provided stability, consistency and droplets’ homogeneity (Gilbert et al., 2013). Our results agree well with those of Estanqueiro et al. (2016) who demonstrated that by increasing the amount of Carbopol®, the droplets’ size decreases, thus, favoring a monodisperse microstructure for the oil droplets.

The emulsions prepared with different concentrations of the MNPs-PEG8-BUF-II-Ag nanobioconjugates exhibited a polydisperse droplets distribution ranging from 1 µm to 10 μm, while those in their absence showed a normal distribution with a particle size of 1.240 ± 0.001 µm (Figure 3B). This polydispersity also provides further evidence for the instability phenomena observed for the emulsion with 400 μg/ml of MNPs-PEG8-BUF-II-Ag nanobioconjugates, mainly because the greater the oil droplets the greater the attractive forces between them, which favors coalescence or flocculation processes. Nonetheless, the particle size of these emulsions (3.950 ± 0.005 µm) remains below that of the commercial treatment (∼35 µm). This was confirmed by observation under optical microscope (100X) (Supplementary Figure S4). Importantly, emulsions with the MNPs-PEG8-BUF-II-Ag nanobioconjugates and specifically those prepared at 400 μg/ml showed substantial polydispersity.

### 3.4 Rheological characterization of O/W emulsions

**FIGURE 4:**
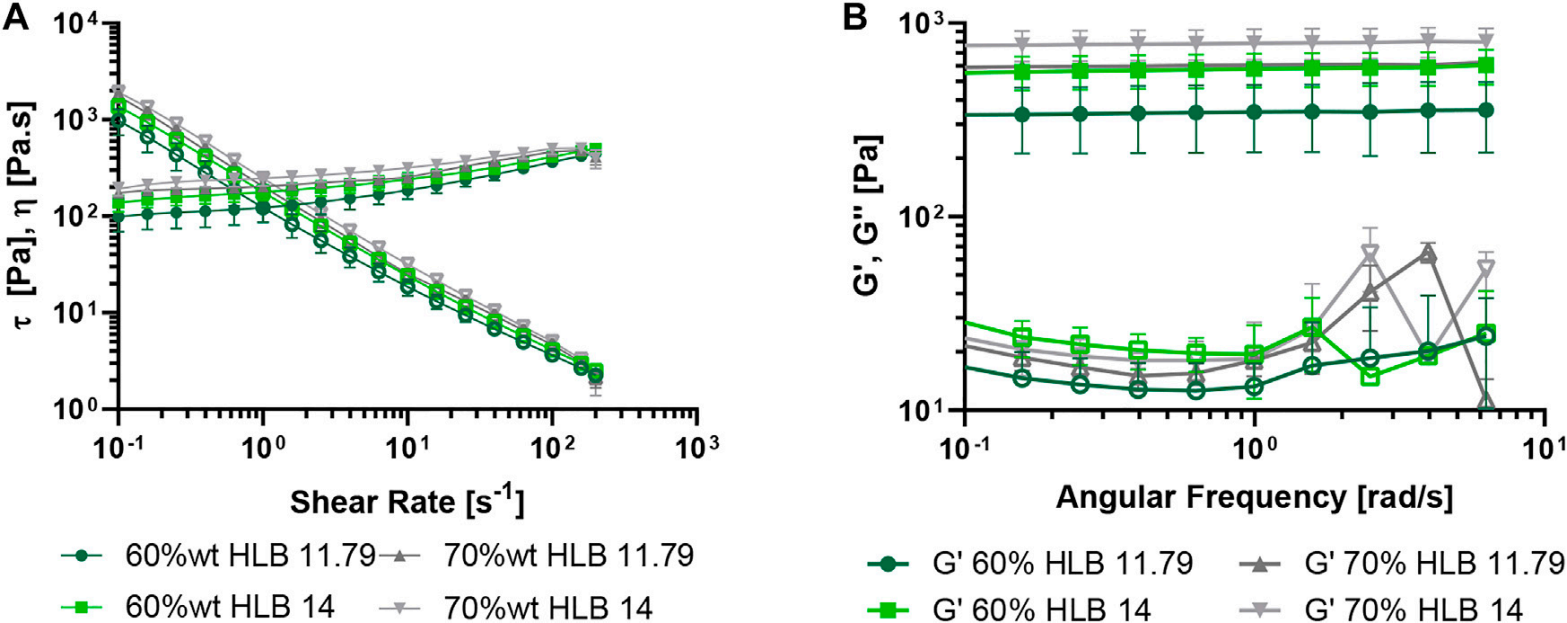
Rheological analyses of O/W emulsions at 20°C. The emulsions were prepared with the MNPs- PEG8-BUF-II nanobioconjugates at 60% wt., 70% wt., HLB of 11.79 and 14. **(A)** Shear stress *τ* vs. shear-rate and viscosity *η* vs. shear-rate (hollow symbols) at 0.1–200 s^−1^. **(B)** Frequency sweep analyses of O/W emulsions performed at 20°C, 1% strain and 0.01–1 Hz (hollow symbols correspond to G″).

O/W emulsions at both dispersed phase concentrations and HLB values evaluated, exhibited a pseudoplastic and a shear-thinning behavior (Figure 4A). The collected rheological data for each emulsion showed that their viscosity increased as a function of the dispersed phase concentration and the HLB value. Regarding the oscillatory sweep test (Figure 4B), the emulsions exhibited a plateau zone where the storage moduli (G′) remained linear for all the emulsions, which indicated the predominance of an elastic response. Even though the G′ value was within the same order of magnitude (∼10 Pa) for all emulsions, the higher the concentration and the HLB value, the higher the storage moduli (Figure 4A). On the contrary, the loss moduli (G″) showed a non-linear behavior (Table 2), with variations of about an order of magnitude between samples. These results indicate that the emulsion will be easily spread over the skin, and it will remain on the tissue because it recovers its viscosity after the application of shear stress (thixotropic character), which was also observed for the commercial topical treatment.

**TABLE 2:** Rheological parameters of bare O/W emulsions at two different concentrations of dispersed phase and two different values of HLB. The values of m and n were recovered from the power law. R2 is the correlation coefficient for data fitting to the power law. While G′ and G′′ correspond to the mean values of each modulus and the corresponding standard deviations.

Rheology indexes and modulus						
Emulsion	R2	M	N	G′	G′′	
60% wt. HLB 11.79	0.957	±131.45	±0.206	±344.23	±16.825	±
	0.217	35.07	0.036	7.194	3.881	
60% wt.	0.953	±182.49	±0.164	±573.26	±23.372	±
HLB 14	0.186	31.47	0.014	17.82	6.057	
70% wt. HLB 11.79	0.881	±213.37	±0.135	±602.37	±25.058	±
	0.383	10.06	0.003	11.40	15.73	
70% wt.	0.872	±252.76	±0.120	±782.31	±28.22	±
HLB 14	0.698	35.95	0.012	14.22	15.82	

**FIGURE 5:**
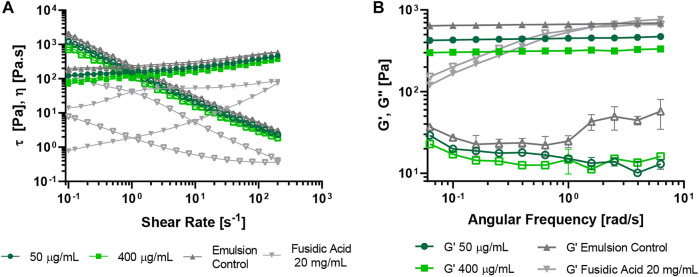
Rheological analysis of O/W emulsions performed at 20°C. The emulsions were prepared with the MNps-PEG8-BUF-II-Ag nanobioconjugates (50 μg/ml and 400 μg/ml) at 60% wt. and HLB 14. The bare emulsion and a commercial control based on fusidic acid were employed as controls. **(A)** Stress *τ* vs. shear-rate and viscosity *η* vs. shear-rate (hollow symbols) at 0.1–200 s^−1^. **(B)** Frequency Sweep analysis of O/W emulsions performed at 20°C, 1% strain and 0.01–1 Hz (hollow symbols correspond to G″).

Considering that the most stable emulsion was the formulation at 60% wt. and HLB 14, two (50 μg/ml and 400 μg/ml) different concentrations of MNPs-PEG8-BUF-II-Ag nanobioconjugates were tested to determine whether their presence might alter the viscosity and the rheological behavior of the emulsions. Figure 5A shows that even though there exists a visible decrease in the viscosity and shear-stress at a 400 μg/ml concentration of nanobioconjugates, the pseudoplastic and shear-thinning behavior remain largely unaltered. This agrees well with previous reports by Sharkawy et al. (2021) and Bhagavathi Kandy et al. (2018) who demonstrated that emulsions containing nano-systems, such as chitosan nanoparticles and multiwalled carbon nanotubes, preserve their pseudoplastic behavior. This distinct elastic and viscous behavior is also related to the presence and the type of the thickening agent (Chhabra and Richardson, 2008; Estanqueiro et al., 2016) and by the high concentration of the oil phase (Li et al., 2018; Calvo et al., 2020).

Regarding the oscillatory sweep, it was observed that both G’ and G” moduli decrease when increasing the concentration of MNPs-PEG8-BUF-II-Ag nanobioconjugates in the continuous phase; however, the elastic modulus is the predominant one (Figure 5B; Table 3). This indicates that the emulsion will not flow rapidly unless an external force is applied. Similar results have been reported previously by Gilbert et al. (2013) for emulsions prepared with synthetic thickeners such as Carbopol® and polyacrylamide. They demonstrated that the viscoelastic behavior can be attributed to the ability of the synthetic textural agents to form gel structures, which improves the resistance of emulsions to structural breakdown (Gilbert et al., 2013). Such behavior was also observed by us in the presence of Carbopol® but was different from that with xanthan gum (Supplementary Figure S5), where much more fluid emulsions were obtained as evidenced by their higher sensitivity to shear-rates and viscosity and moduli values an order of magnitude lower (Supplementary Figure S5B). This was also consistent with previous reports by different authors (Gilbert et al., 2013; Lauterbach and Mu¨ller-Goymann, 2014; Estanqueiro et al., 2016).

**TABLE 3:** Rheological parameters of O/W emulsions (60% wt. and HLB of 14) at two different concentrations of the MNPs-PEG8-BUF-II-Ag nanobioconjugate. The values of m and n were obtained from the power law. While G′ and G′′ correspond to the mean values of each module and the corresponding ^standard deviations.^

Rheology indexes and modulus						
Emulsion	R2	m	n	G′	G′′	
50 μg/ml	0.942	±161.21	±0.1667	±445.35	±16.863	±
	0.007	4.96	0.003	2.222	0.754	
400 μg/ml	0.95 ± 0.000	115.48	±0.1889	±314.19	±14.922	±
		0.12	0.003	2.562	0.204	
Control	0.933	±235.98	±0.1480	±663.76	±34.073	±
	0.009	5.58	0.001	13.43	2.888	
Fusidic Acid	0.4724	7.7221	0.4094	446.67	455.89	

### 3.5 Scaling-up of the emulsion synthesis to the bench scale

**FIGURE 6:**
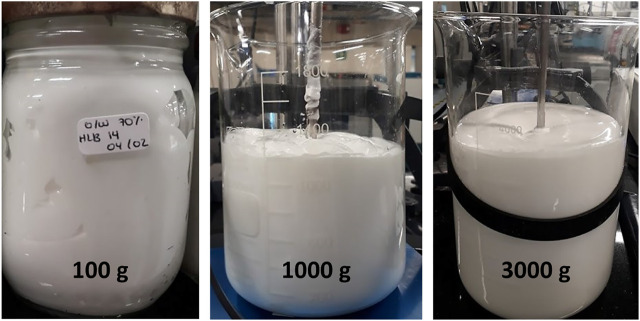
Images of O/W emulsions prepared at 70%wt. and HLB of 14. The experiments were conducted at three different scales: from left to right: 100 g, 1,000 g and 3,000 g.

Considering the results presented previously, the direct emulsion at 60% wt. and HLB 14 was selected for scaling-up from laboratory to bench scale. For this purpose, we explored the synthesis of three different amounts of emulsions (i.e., 100 g, 1,000 g and 3,000 g) (Figure 6). This was accomplished by taking into account several parameters such as the impellers diameters relation (de Oliveira, 2009), the final height of the emulsion contained in the mixing container, and the impeller clearance (see Supplementary Equation S1) (Pradilla et al., 2015; Russell et al., 2019). As part of the scaling-up process, it was also necessary to design the impeller geometry (Supplementary Figure S2) according to the appropriate specifications such as the diameter and the blades height. This was accomplished by keeping the tip velocity (which refers to the velocity that the particles experienced in the tip of the impeller (Sopó et al., 2014; Pradilla et al., 2015)) constant to ensure that the oil droplets were incorporated under the same conditions.

The scaling-up of processes involving Newtonian fluids rely on keeping constant non-dimensional numbers, such as the Reynolds (Re) and Froude (Fr) numbers to obtain the dimensions and conditions of the scaled-up systems (de Oliveira, 2009). However, for non-Newtonian fluids, such as the topical emulsions developed here, the viscosity changes with the shear rate, which makes the estimation of non-dimensional numbers impractical (Chhabra and Richardson, 1999; de Oliveira, 2009). This can be addressed by calculating the non-dimensional numbers through empirical equations that include rheological indexes (Chhabra and Richardson, 1999; Tadros, 2013). Alternatively, a more accurate scaling-up parameter is the tip velocity due to its independency with respect to the rheology of the fluid (Pradilla et al., 2015; Reyes and Ratkovich, 2018a). Regarding the impeller selection, it is necessary to consider its pumping capacity (widely reported for different geometries) (Chhabra and Richardson, 1999; Reyes and Ratkovich, 2018a; Reyes and Ratkovich, 2018b), and the type of flow produced (i.e., axial or radial) because these parameters largely define the vortex formation and the obtained flow regime (Chhabra and Richardson, 1999; Russell et al., 2019).

A flow sweep experiment for the three formulations obtained at a larger scale showed that in all cases a pseudoplastic and shear-thinning behavior was observed; however, the consistency and flow indexes decreased as the amount prepared increased (Supplementary Figure S6A). This phenomenon was also observed in the oscillatory tests where, despite the linearity of G′ modulus for the three preparations, the value of the modulus decreased with the amount of emulsion prepared. The G″ modulus showed no clear trend for the different samples (Supplementary Figure S6B). The significant differences observed for the 3,000 g sample can be attributed to changes in the vessel geometry and dimensions, and most likely to the height/diameter (T/H) ratio for the tank that approached ∼0.65; however, previous reports have recommended a 1 ratio to avoid dead zones.

Regarding the droplet size, the distribution of the 1,000 g and 3,3000 g preparations is similar with D [4,3] values of 1.25 µm and 1.21 µm, respectively (Supplementary Figure S7A). Despite the differences in the volume density percentage with respect to the 100 g preparation, the obtained droplet sizes remained at about the same order of magnitude. This confirms that the performance of the scaled-up systems is comparable to that at the lab scale (Rawle et al., 2003). Regarding the final product properties, the emulsion prepared at the bench scale exhibited a lower viscosity leading to sensorial differences when compared with the emulsion prepared at the laboratory scale. In addition, stability in time may vary from one scale to the other, most likely due to the differences in pumping performance during the mixing process. For this reason, it is important to maintain the scaling-up parameters and vessel dimensions and consider including mixing enhancing accessories like baffles to assure sufficient shear stress and turbulence. This could help to avoid differences or variation of properties like spreadability, retention time and stability between the different production scales.

### 3.6 Antibacterial assay

In previous studies, Perez et al. (2019) synthesize MNPs-PEA-BUF-II nanobioconjugates and identified internalization in both prokaryotic (*E. coli*) and eukaryotic (HaCaT, THP-1) cells, as well as the capability of avoiding endocytic internalization pathways in several animal and human derived cell lines. However, the MNPs-PEA-BUF-II nanobioconjugates (1 mg/ml) antibacterial action leads to a reduction of barely ∼50% of bacterial growth. With the aim of boosting the antibacterial effect of such nanobioconjugates, it was necessary to replace the PEA spacer by longer heterobifunctional PEG (PEG8). Also, a silver shell was synthesized on the surface of the new MNPs-PEG8-BUF-II nanobioconjugates to increase their antibacterial capacity even further. Despite BUF-II’s lower antibacterial activity compared with silver, it was maintained on the nanobioconjugates because it increases their aqueous stability and likely enhances their biocompatibility (see Supplementary Figures S8A,B). This was evidenced by the superior biocompatibility and stability of the obtained emulsions, which has been reported as one of the main challenges in the preparation of products based on antimicrobial peptides (Mun˜oz-Camargo et al., 2018). The emulsion with MNP-PEG8-BUF-II-Ag nanobioconjugates (400 μg/ml) was assessed against skin secretions infected with methicillin resistant strains of *S. aureus* (MRSA clinical isolates 92250621, 93040389 and 93190573) and reference ATCC strains of *S. aureus* and *E. coli*.

**FIGURE 7:**
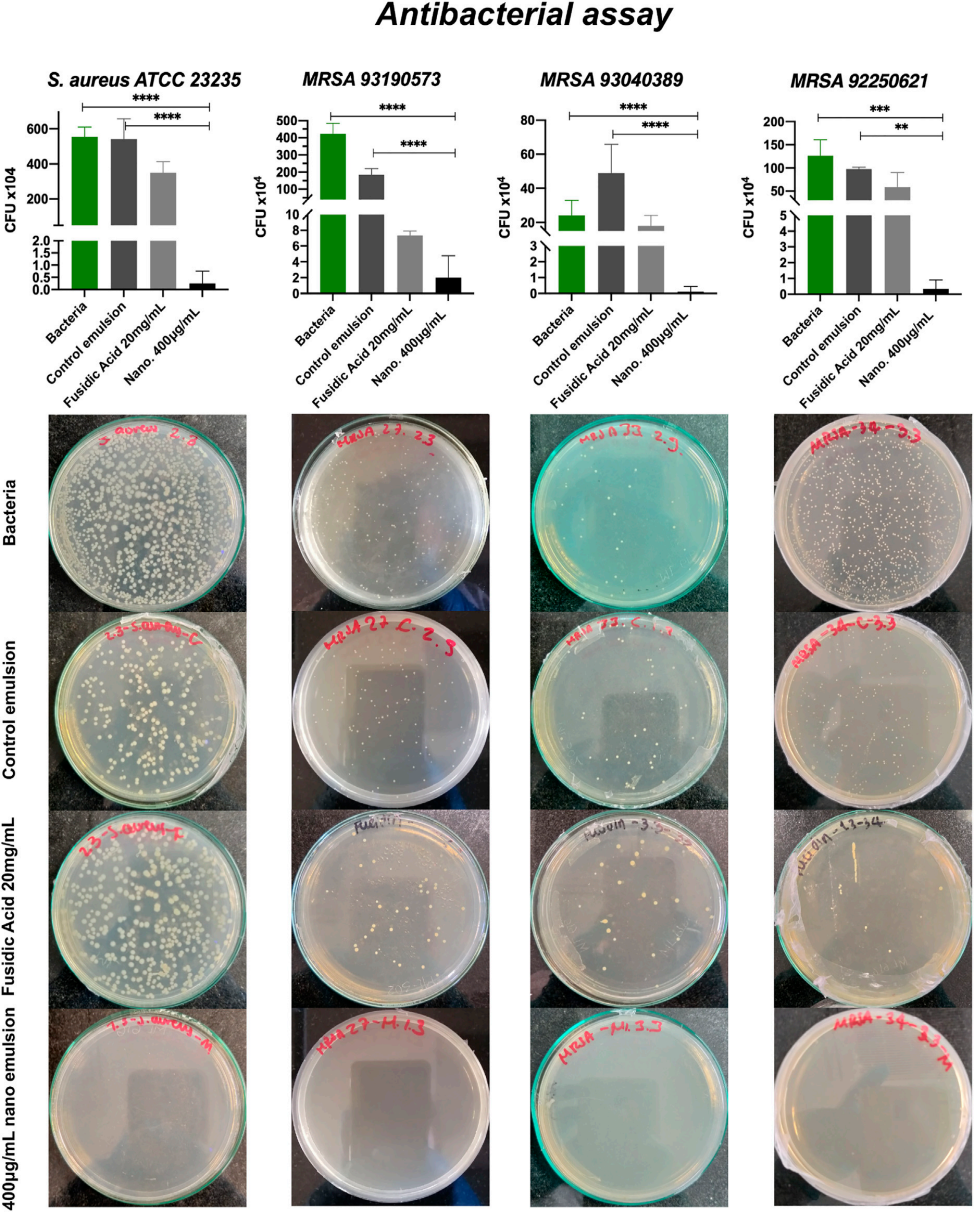
Antibacterial activity assay performed with *S. aureus* (ATTC 23235) and MRSA clinical isolates (92250621, 93040389 and 93190573). The emulsion tested was prepared with the MNP-PEG8-BUF-II-Ag nanobioconjugates (400 μg/ml) at 60% wt. and HLB of 14. The emulsion prepared in the absence of the nanobioconjugates and a commercial topical based on fusidic acid (20 mg/ml) were employed as controls in the assay. The third dilution (1 × 104 CFU) was seeded. The positive control was bacteria grown in Na2HPO4 buffer. (****) corresponds to *p <* 0.0001, (***) to *p <* 0.001 and, (**) *p <* 0.01. Statistically significant differences were for *p <* 0.05.

Figure 7 shows that the formulated emulsion (400 μg/ml of the MNPs-PEG8-BUFF-II-Ag nanobioconjugates) significantly (*p <* 0.0001) inhibited the growth of *93040389* and *93190573 MRSA* and *S.aureus* strains, and that of *92250621 MRSA* strain with a lower statistical significance (*p <* 0.005) compared with the emulsion in absence of the nanobioconjugates. Moreover, the efficacy of the treatment containing the nanobioconjugates was demonstrated by comparing its performance with the commercial preparation containing fusidic acid (20 mg/ml) as active agent. In this regard, our emulsion exhibited a higher inhibitory action than the commercial control in the *MRSA clinical isolates 93190573* and *93040389* and *S. aureus* (ATTC 23235). However, no significant difference (*p >* 0.05) was observed for the *MRSA 92250621*. These promising results can be largely attributed to the synergistic effect of silver and BUF II because while the peptide promotes membrane translocation, silver has been reported to disrupt them very effectively (Park et al., 2000; Mun˜oz-Camargo et al., 2018). In one hand, a previous study involving the AMP C18N3/CARG by Jiang et al. (2022) formed complexes of surfactants with hydrocarbons and the peptide, which led to *β*-lactamase inhibition and consequently, partial *MRSA* growth inhibition (∼50%) in the presence of antibiotics such as ampicillin. On the other hand, Karthik et al. (2021) evaluated silver nanoparticles Bcp*C@AgNPs as an antibacterial agent against *MRSA*, obtaining only a 35% growth inhibition at a concentration of 320 μg/ml. Compared with these two separate studies discussing the potency of silver and an AMP as antibacterial agents, our formulation showed a significantly superior performance. In addition, the control emulsion showed antibacterial effect on *MRSA 93190573* and *MRSA 92250621* and non-antibacterial effect on *MRSA 93040389*. Tween 80, which was a surfactant incorporated into the control emulsion formulation, has been reported to reduce bacterial growth mainly due to its properties of ionophore that allow it to chelate metal ions like *K* ^+^ *, Na* ^+^ and *Ca* ^+^, which are key for numerous cell processes (Nielsen et al., 2016). As a result, enzyme activity and bacterial adhesion might be severely impaired. Considering that MRSA 93190573 and MRSA 92250621 showed a slower growth compared with MRSA 93040389, it is very likely that Tween 80 has a more pronounced effect on such strain. This can be seen in the growth curve (Supplementary Figure S11) carried out after 24 h in LB broth at 37°C (Nielsen et al., 2016).

Additionally, the commercial control containing fusidic acid (20 mg/ml) is marketed as one of the main alternatives to treat cutaneous infections against wild type strains and resistant strains. However, our results clearly indicate that this topical treatment shows antibacterial activity at concentrations that are 2-32 times higher than MIC (minimal inhibitory concentration) (Dobie and Gray, 2004). Moreover, this commercial control has a bacteriostatic activity compared with the strong bactericide activity of our emulsions (Vingsbo Lundberg and Frimodt-Møller, 2013; Bessa et al., 2016). Besides the effectiveness of our topicals for treating MRSA skin infections, we hypothesize that the developed formulations might be useful to treat other infections that involve resistant microorganisms. Taken together, the antibacterial activity results for our topical treatment indicate that it holds much promise as a novel alternative to mupirocin- and fusidic acid-based formulations to combat nosocomial and community acquired infections (Elston, 2009; Bessa et al., 2016; Watkins et al., 2019).

### 3.7 Qualitative wound infection assay *ex vivo*

A first approach to test the effectiveness of the topical treatment in an infected wound was executed *ex vivo* in porcine skin. Two different infection times were evaluated to determine the incubation time that mimics *in vivo* infected tissues the closest. Supplementary Figure S9 shows color changes around the exposed tissue area to 1 × 10^7^ CFU at 24 h and 48 h, which may indicate the bacterial growth upon inoculation. Nonetheless, the infection in the sample treated on day 0 was more noticeable and seemed to be more homogeneous than the sample infected 24 h after incubation. Importantly, the tissue exhibited an intrinsic color change and general appearance most likely due to the temperature conditions of (37°C), and the lack of cell culture media for the maintenance of the keratinocytes and fibroblasts present in the skin tissue. Additionally, 24 h after the application of the topical treatment, the emulsion diffused along the infected tissue, which indicates that the nanobioconjugates dispersed in the emulsion will most likely reach the proliferated bacteria cells and, therefore, reduce or completely inhibit their viability. This assay allowed us to conclude that further studies should be conducted in an infected wound prior to incubation and that the treatment 72 h after the infection. Additionally, with the rapid emergence of additive biofabrication techniques, further assessment will consider the use of a 3D bioprinted human skin model that offers a more reliable platform for the study of the interaction of bacteria and the emulsions as it recapitulates much more closely the physiology of the skin without the bioethical issues of animal models.

As has been reported previously by Raveendran et al. (2019) and Rosselle et al. (2020), among many others (Nataraj et al., 2014; Singh et al., 2019; Currie et al., 2020), wound infected assays performed in pigskin provide preliminary data of not only the antibacterial activity of novel treatments but also about possible additional undesirable effects on the tissue. These experiments allowed the authors to evaluate different skin treatments prior to *in vivo* experiments aided by qualitative macroscopic analysis and advanced microscopy techniques.

### 3.8 Biocompatibility assays

#### 3.8.1 Hemolysis and platelet activation assays

Considering that the topical formulation is intended for the treatment of skin’s surface infections, the tests were performed without diluting the emulsion. Preliminary results of diluted topicals ranging from 50% (w/v) down to 1.65% (w/v) indicated hemolysis percentages below 5% (data not shown). However, platelet aggregation varied from 50% to 80% at some of the evaluated concentrations, and consequently. Platelet activation was tested instead.

**FIGURE 8:**
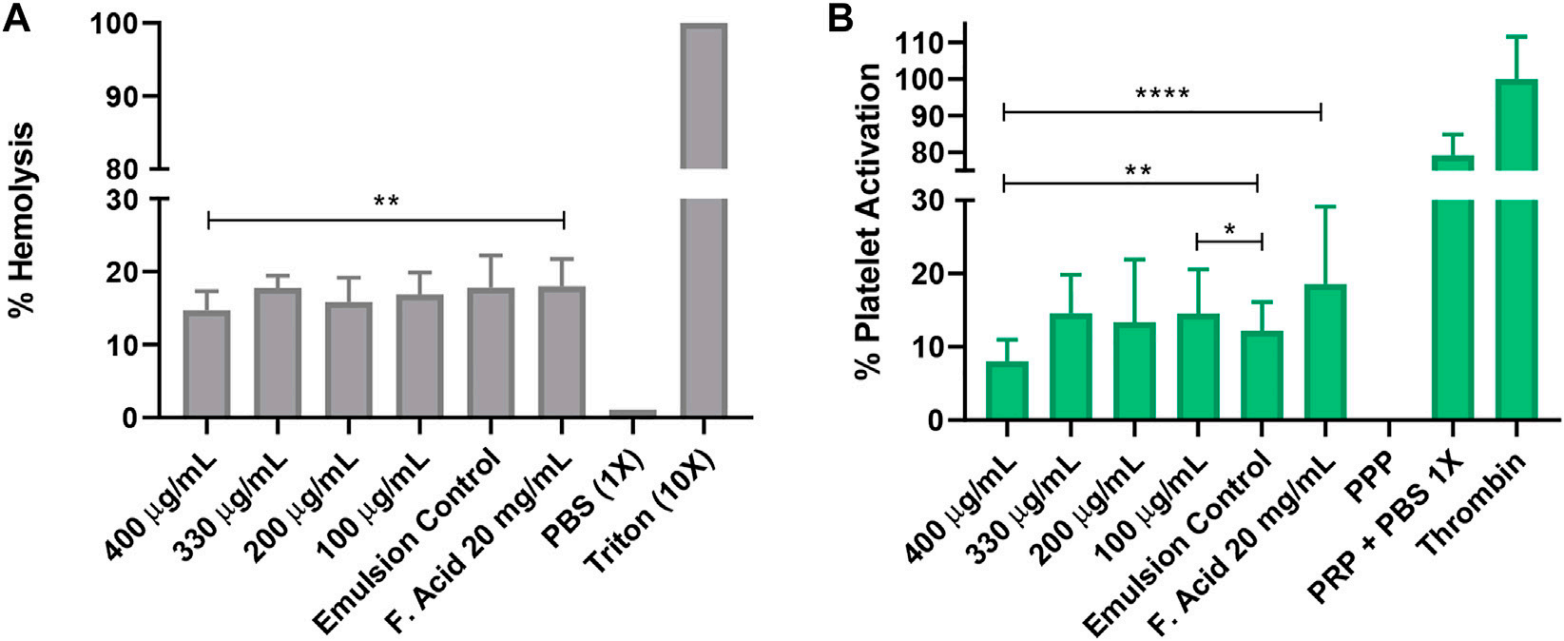
Biocompatibility assays. The tested topicals were prepared with the MNPs-PEG8-BUF-II-Ag nanobioconjugates (400, 330, 200 and 100 μg/ml) at 60% wt. and HLB of 14. The emulsion prepared in the absence of the nanobioconjugates and a commercial topical based on fusidic acid were employed as controls. In the hemolysis assay **(A)** Triton X-100 was used as the positive control, while PBS 1X was the negative one. In the platelet activation assay **(B)** plasma poor in platelets (PPP) was employed as the negative control and Thrombin as the positive one (****) corresponds to *p <* 0.0001, (***) to *p <* 0.001, (**) to *p <* 0.01 and, (*) *p <* 0.05. Statistically significant differences were for *p <* 0.05.

Figure 8A shows that the O/W emulsion with different concentrations of the MNP-PEG8-BUF-II-Ag nanobioconjugate (400, 330, 200, and 100 μg/ml) exhibited a hemolysis percentage ranging from 15% to 20%. Similar results were obtained for the bare emulsions and for the commercial treatment Fucidin®. However, there was no statistical difference between our emulsion at 400 μg/ml of the MNP-PEG8-BUF-II-Ag nanobioconjugate and the commercial control of Fusidic Acid 20 mg/ml (*p* = 0.0094). Despite obtaining a hemolysis percentage above 5% (which is the limit indicated in the ISO 10993-4 standard to consider that a medical device is not hemolytic (Standard, I., 2002)), the hemolysis percentage obtained is acceptable considering that the products are not intended for open wound treatment (Organisation for Economic Co-operation and Development 2018; Organisation for Economic Co-operation and Development 2021).

Figure 8B shows that the platelet activation ranged from 10% to 20% for all the tested treatments. This interesting result indicates that during the treatment against the MRSA infection, the topicals might be concomitantly able to support and improve the healing process (Eisinger et al., 2018; Wilkinson and Hardman. 2020).

#### 3.8.2 Cytotoxicity assay

**FIGURE 9:**
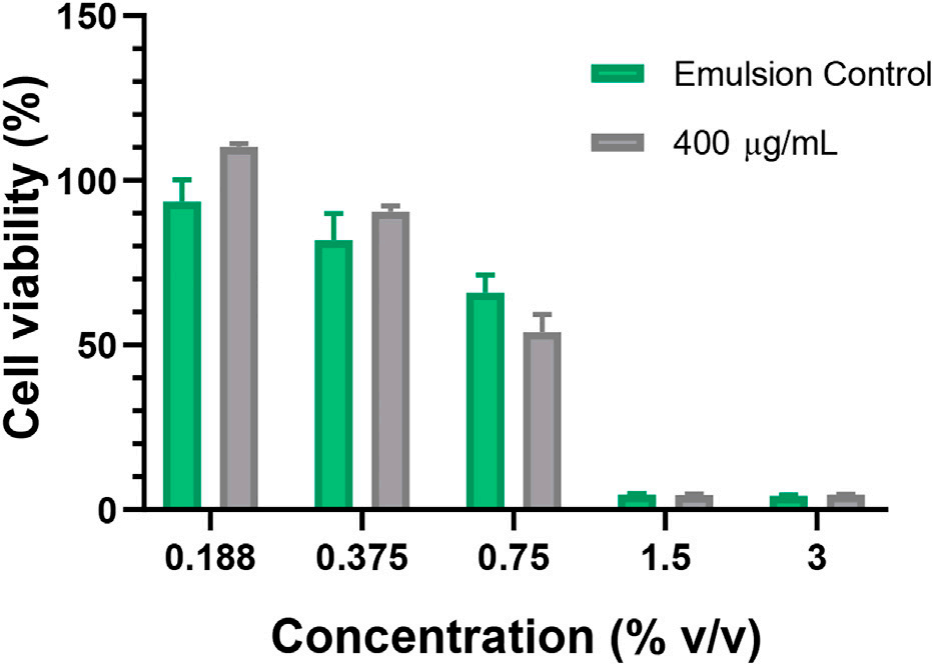
Cytotoxicity assay. The tested emulsion was prepared with the MNPs-PEG8-BUF-II-Ag nanobioconjugates (400 μg/ml) at 60% wt. and HLB of 14. The emulsion prepared in the absence the nanobioconjugates was employed as control.

The cytotoxic effect of the emulsions was assessed through an indirect assay based on exposing cells to their extracts, Figure 9 indicates that at concentrations above 1.5% (v/v) both emulsions are highly cytotoxic. This result may be related to the high oil content of the emulsions, which prevents them to fully dissolve in aqueous buffers and therefore a high amount of such components tends to accumulate at the surface of the solution. As a result, when cells are exposed to the treatments, the formed oil layer impedes the appropriate gas exchange. Which is detrimental to cells survival (Supplementary Figure S10). Our cell viability results are comparable with those of Okur et al. (2020); Coelho De Bari et al. (de Bari et al., 2016) and Płaczek et al. (2019) who developed therapeutic emulsions for topical. Oral and parenteral administration. The authors reported cell viability of murine or human cell lines above 70% when exposed to emulsions at concentrations below 4% (v/v).

Emulsions are widely used in the pharmaceutical field. However those with high viscosity, like creams, have found application in skin cancer treatment due to their versatility to incorporate active agents and various excipients (Cullen et al., 2020). Also, when essential oils and surfactants (such as polysorbate 80) are used to prepare them, they tend to exhibit significant cytotoxicity towards cancer cells and tumors (Russo et al., 2015; de Bari et al., 2016). Despite reducing cell viability of 3T3 cultures, polysorbate 80 has been incorporated into several skin care commercial products, body lotions and shampoos. Moreover, some antibacterial topical treatments contain low concentrations of this component combined with other molecules to improve their colloidal stability (Vanangamundi. 2010).

## 4 Conclusion

Antibiotic-resistant bacteria pose a major challenge for the healthcare systems worldwide. Big pharma companies have reduced budgets dedicated to finding alternatives to conventional treatments. Novel approaches based on peptides and functionalized nanomaterials appear to provide a route for the cost-effective production of more potent antibacterial formulations. Here, we explored such an approach in the design and preparation of topical formulations for treating skin infections by Methicillin-resistant *Staphylococcus aureus* (MRSA). The topicals were based on O/W emulsions supplemented with dispersed MNP-PEG8-BUF-II-Ag nanobioconjugates as active antibacterial agents. The prepared emulsions were characterized physicochemically by rheological assays, particle size and stability measurements. and microscopy imaging. Superior characteristics and colloidal stability were maintained even after scaling the synthesis process to 1,000 and 3,000 g. The formulated topicals also demonstrated high antibacterial activity against several MRSA strains. Furthermor, results of hemolysis and platelet aggregation assays were in the range of those exhibited by the commercial control, which is critical prior to move to *in vivo* testing. Finally, the bactericidal effect of the topical treatment was evaluated *ex vivo* over an infected wound in a porcine skin cut.

Future work will be dedicated to evaluating the emulsions in a 3D human skin model where co-infection will be recreated and then tested *in vivo*.

## Data Availability

The original contributions presented in the study are included in the article/Supplementary Material, further inquiries can be directed to the corresponding author.
