# Supplementary material for: Formulation of a novel antibacterial topical treatment based on Magnetite-Buforin-II-silver nanobioconjugates
Source: Front Bioeng Biotechnol. 2022 Oct 28;10:1003004. doi: 10.3389/fbioe.2022.1003004 (PMC9649911; doi:10.3389/fbioe.2022.1003004)
Supplement: Supplementary file 1 [file DataSheet1.pdf]

Supplementary Material

1 SUPPLEMENTARY TABLES

Table S1. Parameters and dimensions used in the scaling-up process to reach three different scales (100 g, 1000 g and 3000 g).

			Scale-up Process Dimensions [cm]		
	**Abbreviation**	**Parameter**	**100g**	**1000g**	**3000g**
2[1]*Recipient	T	Inner Diameter	6,62	12,90	16,47
	H	Height	9,98	19,30	27,00
7[2]*Impeller	D	Total Diameter	5,30	9,40	13,18
	L	Blade Length	1,77	4,30	5,58
	W	Blade Height	2,02	2,15	2,79
	B	Width	0,30	0,40	0,42
	A	Axis Width	3,64	6,40	6,40
	S	Shaft Diameter	7,74	8,20	8,20
	°	Angle of Declination	60	60	60
2[2]*Assembly	C	Clearance	2,65	5,16	6,59
	Z	Liquid Height	6,62	12,90	16,47

The inner diameter (T) and the height (H) of the recipient were measured in the laboratory.

The impeller diameter (D), the blade height (W), the liquid height (Z) and the clearance (C) were calculated with the following relations:

$$D/T = 0,8,\ W/L = 0,5,\ Z/T = 1,\ C/T = 0,4 \quad \text{(S1)}$$

2 SUPPLEMENTARY FIGURES

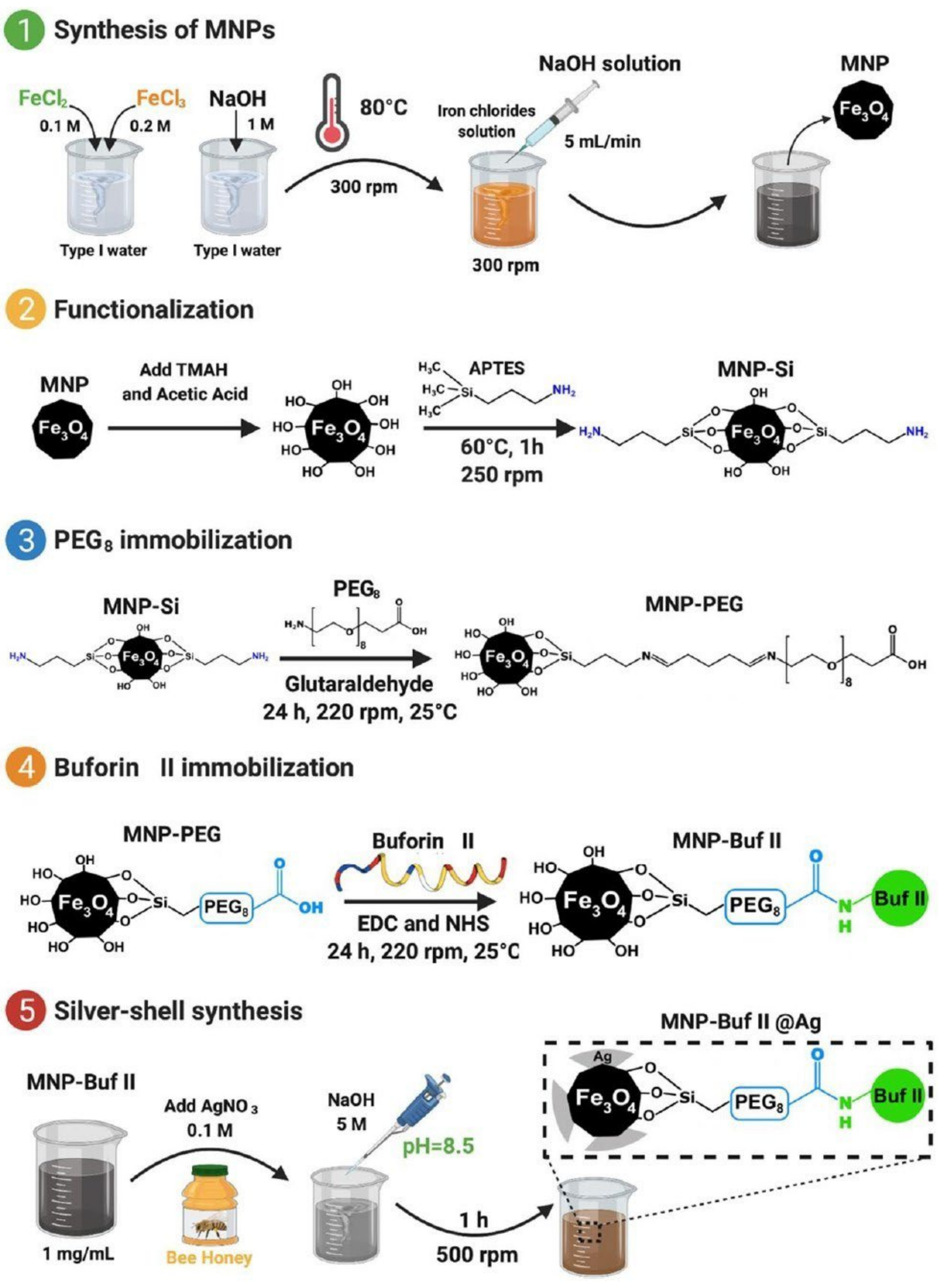

Figure S1. Protocol and reactions involved in the synthesis of the MNP-BUF-II-Ag nanobioconjugates.

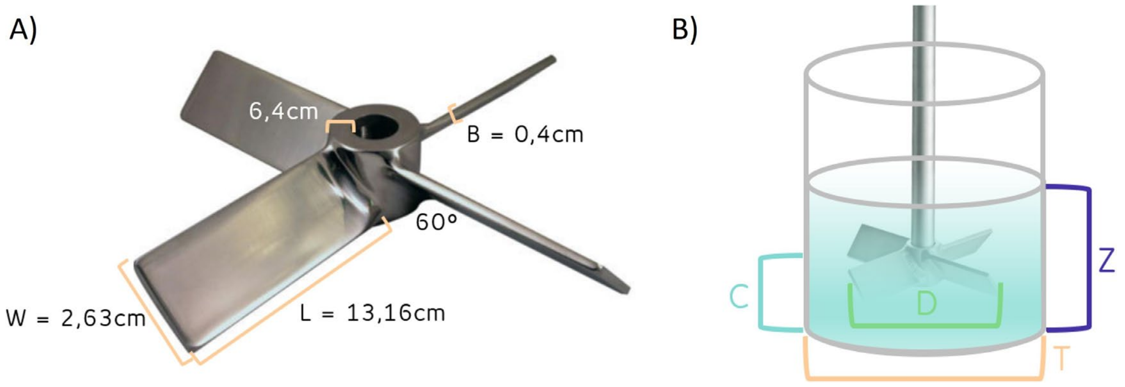

Figure S2. Scaling-up assembly. A) 60° pitch-blade impeller dimensions employed in the scaling-up process (3000 g). B) Impeller location in the vessel.

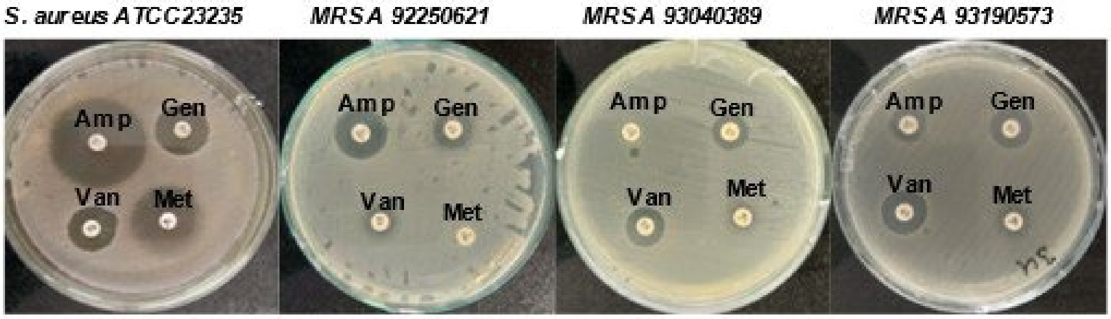

Antibiotic	Inhibition standard (mm)	*S. aureus ATCC 23135*	*MRSA 92250621*	*MRSA 93040389*	*MRSA 93190573*
Methicilin	<9 = R, 10-13 = I, >13 = S	S	R	R	I
Gentamicin	<13 = R, 13-14 = I, >14 = S	S	S	I	S
Vancomycin	>15 = S	S	R	S	S
Ampicillin	<28 = R, >29 = S	S	R	R	R

Figure S3. Antibiograms obtained for wild type *S. aureus* and MRSA strains against methicillin (5 µg), ampicillin (10 µg), gentamicin (10 µg) and vancomycin (30 µg). S. aureus ATCC23235 is susceptible to all antibiotics. MRSA 92250621 is resistant to methicillin and ampicillin and susceptible to gentamicin. MRSA 93040389 is resistant to methicillin and ampicillin, intermediate for gentamicin and susceptible to vancomycin. MRSA 93190573 is resistant to ampicillin, intermediate to methicillin and susceptible to gentamicin and vancomycin.

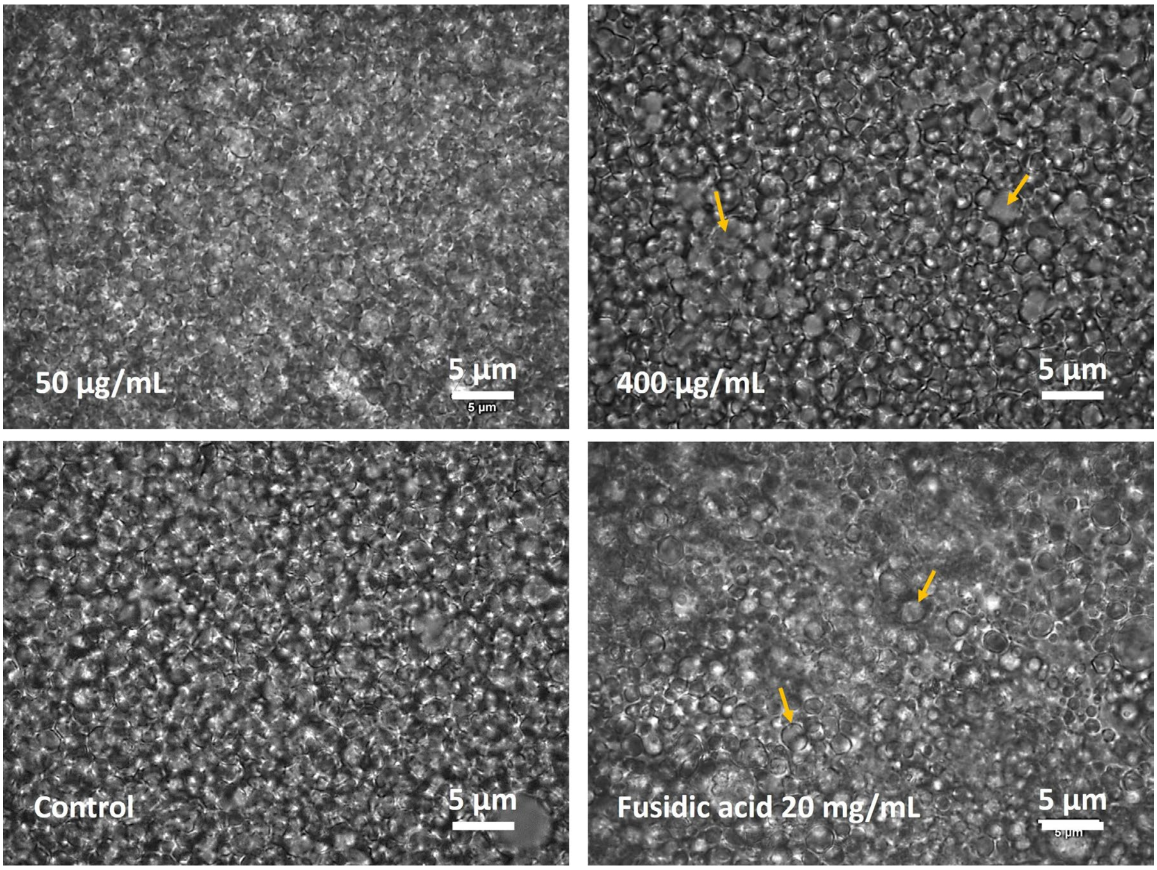

Figure S4. Optical microscope observation, 100X (scale bar 5µm), of the O/W emulsions at 60% wt. and HLB of 14 prepared with the Mag-PEG8-BUF-II-Ag nanobioconjugates (50 µg/mL and 400 µg/mL) 90 days after their preparation. Orange arrows point to larger droplets.

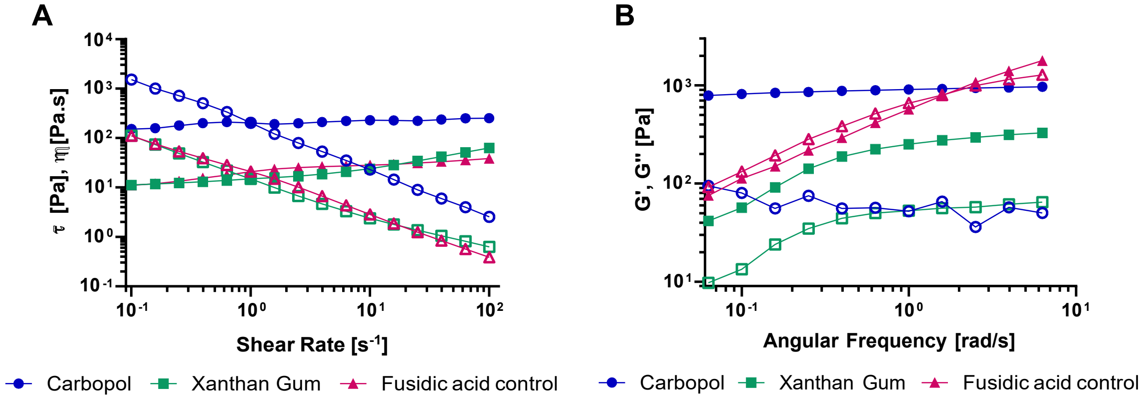

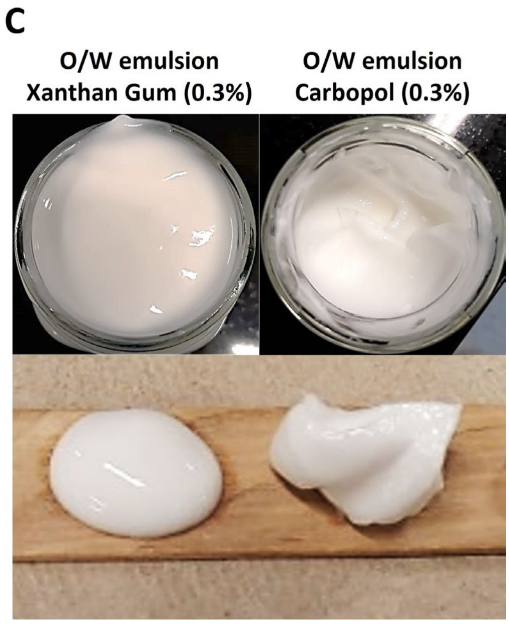

Figure S5. Rheology of O/W emulsions (60% wt.) prepared with two different thickeners: Carbopol® and Xanthan gum. A) Shear stress σ (solid symbols) and viscosity η (hollow symbols) performed at 20°C, 0.1 - 100 s-1. B) Frequency Sweep analysis at 20°C, 1% strain and 0.01 - 1Hz. Storage modulus G" (solid symbols) and loss modulus G' (hollow symbols). C) Images of the O/W emulsions containing either Carbopol® or Xanthan gum as thickener. In these images, the emulsion prepared with Xanthan gum seems more fluid than the one with Carbopol®.

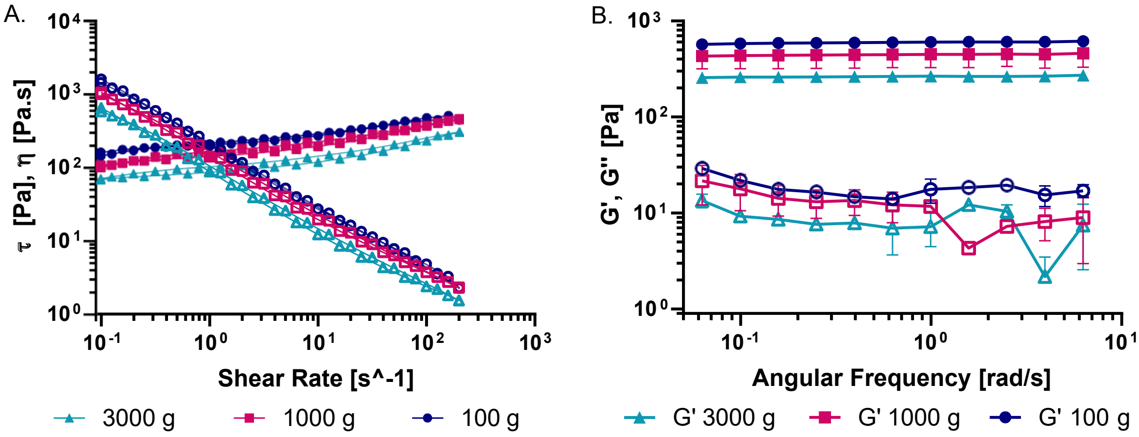

Figure S6. Rheology of O/W emulsions prepared at three different volume scales at 60% wt. and HLB of 14. A) Shear stress σ (solid symbols) and viscosity η (hollow symbols) performed at 20°C, 0.1 - 200 s-1 and 200 – 0.1 s-1. B) Frequency Sweep analysis at 20°C, 1% strain and 0.01 - 1Hz.

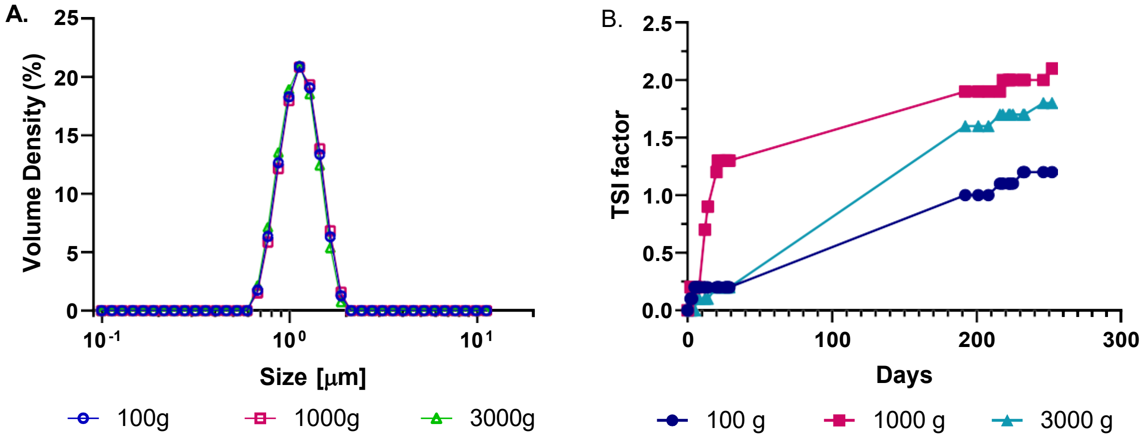

Figure S7. A) Mean hydrodynamic diameter and B) time evolution of stability index for O/W emulsions prepared at different volume scales (100 g, 1000 g, 3000 g).

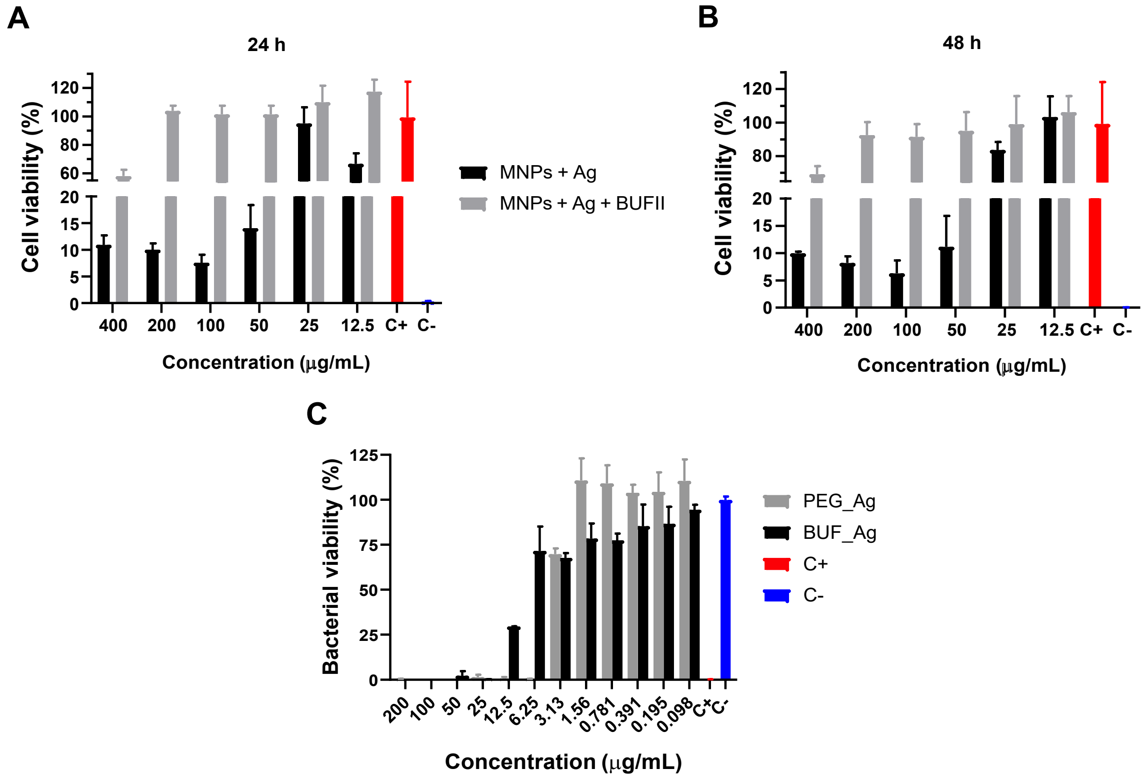

Figure S8. Biocompatibility of the MNPs-PEG8-BUFII-Ag nanobioconjugates. A) Cell viability (Vero cell line) after 24 h of exposure to MNPs-Ag nanoparticles in the presence and absence of the peptide BUF-II. B) Vero Cell viability after a 48 h of exposure to MNPs-Ag nanoparticles in the presence and absence of the peptide BUF-II. C) Antibacterial activity (performed with *S. aureus* (ATTC 23235)) of MNPs-Ag nanoparticles (24 h after exposure) alone or co-immobilized with the peptide BUF-II.

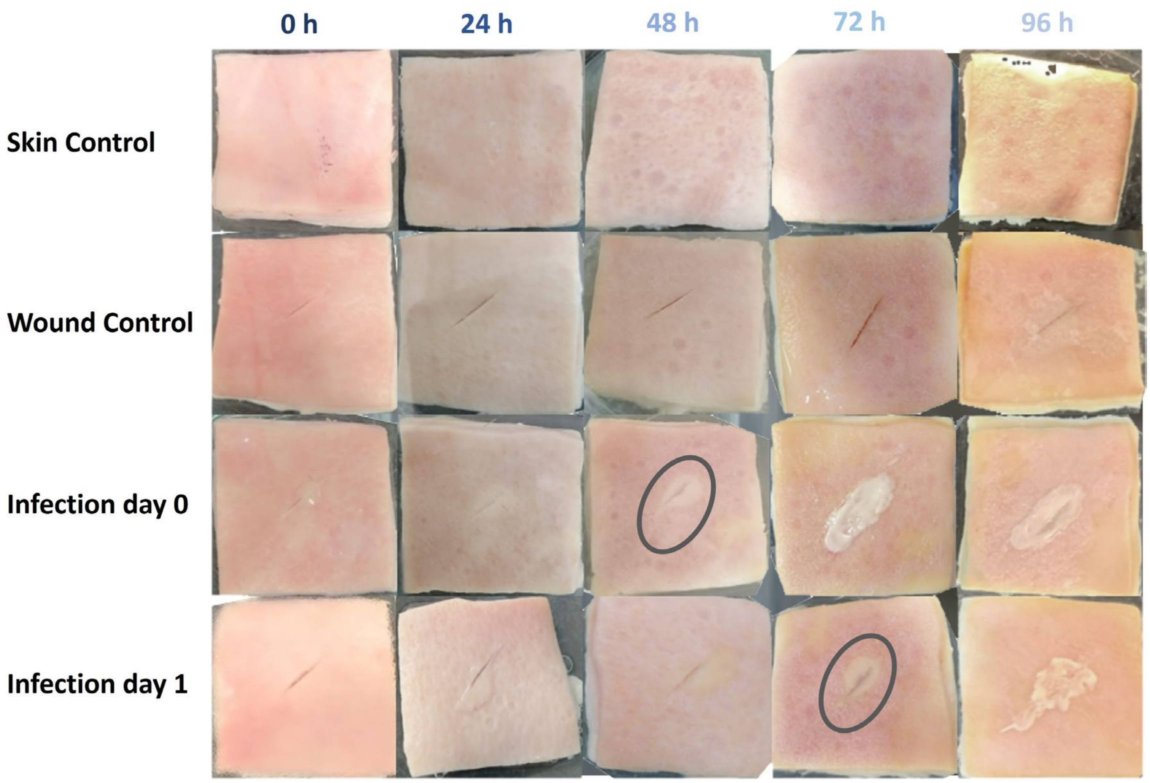

Figure S9. Wound infection performed with *S. aureus* (ATCC 23235 1x107 CFU) on pigskin. Blue circles indicate the infected area 48 h after inoculation. The image in the top panel corresponds to the sample infected at day cero while the image on the bottom one corresponds to the sample incubated 24 h before inoculation.

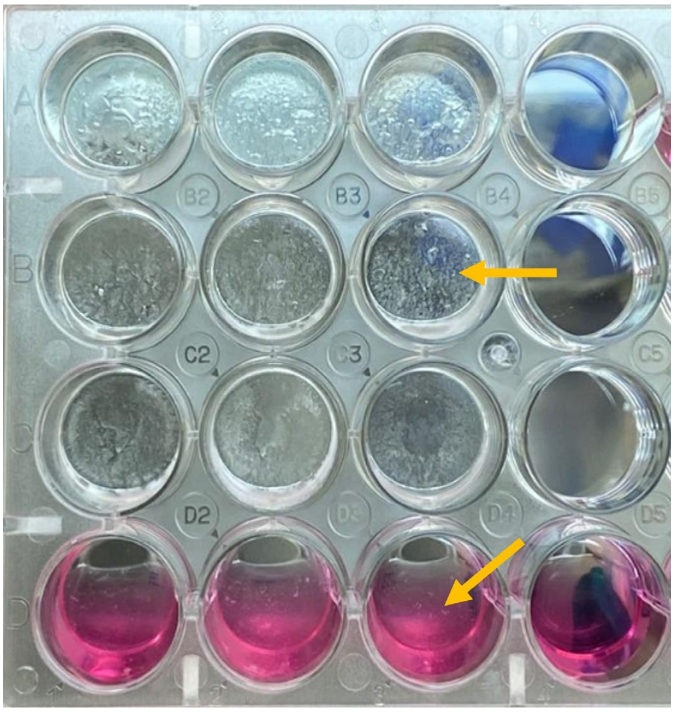

Figure S10. . Image of a 24 well-plate after exposure of HaCaT cells to a 50% v/v solution of the emulsion containing the MNPs-BUF-II-Ag nanobioconjugates (400 µg/mL). The orange arrows indicate the oil phase after removing the DMEM media (upper) and the oil phase on the surface of the DMEM media (down).

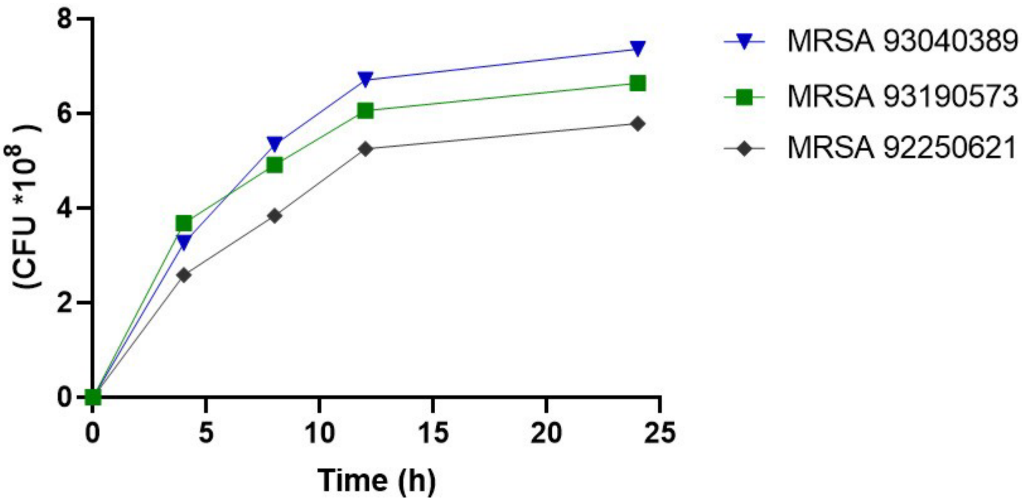

Figure S11. Growth curve for MRSA strains tested prior to antibacterial assays. The strains were grown in LB liquid culture medium with agitation at 37°C for 24 hours

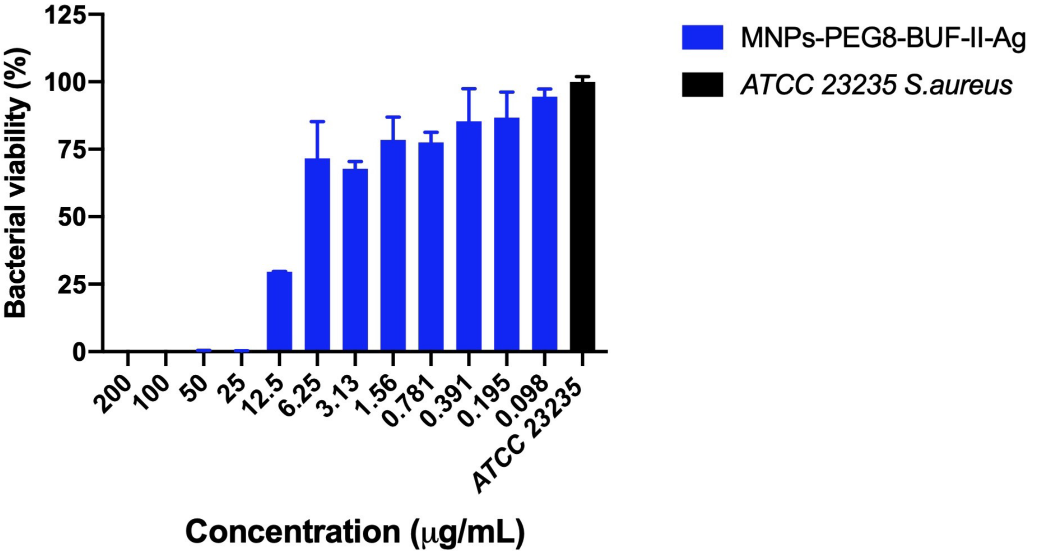

Figure S12. Microdilution bacterial survival test performed using MNP-PEG8-BUF-II-Ag nanobioconjugates (30 h after exposure) against the strain *ATCC 23235 S. aureus*.
